# Comparative Analysis of Secretomes from Ectomycorrhizal Fungi with an Emphasis on Small-Secreted Proteins

**DOI:** 10.3389/fmicb.2015.01278

**Published:** 2015-11-18

**Authors:** Clement Pellegrin, Emmanuelle Morin, Francis M. Martin, Claire Veneault-Fourrey

**Affiliations:** ^1^UMR 1136 Interactions Arbres/Microorganismes, Université de LorraineVandoeuvre-lès-Nancy, France; ^2^UMR 1136 Interactions Arbres/Microorganismes, Laboratoire d'Excellence ARBRE, Institut National de la Recherche Agronomique, INRA-NancyChampenoux, France

**Keywords:** ectomycorrhizal, saprotrophs, secretomics, small-secreted proteins, symbiosis, secretomes

## Abstract

Fungi are major players in the carbon cycle in forest ecosystems due to the wide range of interactions they have with plants either through soil degradation processes by litter decayers or biotrophic interactions with pathogenic and ectomycorrhizal symbionts. Secretion of fungal proteins mediates these interactions by allowing the fungus to interact with its environment and/or host. Ectomycorrhizal (ECM) symbiosis independently appeared several times throughout evolution and involves approximately 80% of trees. Despite extensive physiological studies on ECM symbionts, little is known about the composition and specificities of their secretomes. In this study, we used a bioinformatics pipeline to predict and analyze the secretomes of 49 fungal species, including 11 ECM fungi, wood and soil decayers and pathogenic fungi to tackle the following questions: (1) Are there differences between the secretomes of saprophytic and ECM fungi? (2) Are small-secreted proteins (SSPs) more abundant in biotrophic fungi than in saprophytic fungi? and (3) Are there SSPs shared between ECM, saprotrophic and pathogenic fungi? We showed that the number of predicted secreted proteins is similar in the surveyed species, independently of their lifestyle. The secretome from ECM fungi is characterized by a restricted number of secreted CAZymes, but their repertoires of secreted proteases and lipases are similar to those of saprotrophic fungi. Focusing on SSPs, we showed that the secretome of ECM fungi is enriched in SSPs compared with other species. Most of the SSPs are coded by orphan genes with no known PFAM domain or similarities to known sequences in databases. Finally, based on the clustering analysis, we identified shared- and lifestyle-specific SSPs between saprotrophic and ECM fungi. The presence of SSPs is not limited to fungi interacting with living plants as the genome of saprotrophic fungi also code for numerous SSPs. ECM fungi shared lifestyle-specific SSPs likely involved in symbiosis that are good candidates for further functional analyses.

## Introduction

In forest ecosystems, tree roots are continuously in contact with beneficial, commensal and pathogenic soil microbes. These microbial communities, called the microbiome, are also responsible for nutrient (C, N, and P) recycling and exchanges and have an impact on soil fertility (Chaparro et al., 2012; Lakshmanan et al., 2014) and carbon sequestration (Schimel and Schaeffer, 2012). Consequently, the root microbiome is driving forest health, productivity and sustainability (Wagg et al., 2014). Among those microorganisms, fungi stand as key players that demonstrate a wide range of interactions with plants. This includes wood decayers, saprotrophic soil decomposers, plant pathogens and mutualistic symbionts (Bonfante and Genre, 2010; Veneault-Fourrey and Martin, 2011). Ectomycorrhizal (ECM) symbioses appeared more than 180 Mya (Hibbett and Matheny, 2009) several times independently (Ryberg and Matheny, 2012; Kohler et al., 2015). ECM symbioses evolved from ecologically diverse decayer precursors (white- and brown-rot wood decayers, litter decayers) and radiated in parallel, following the origins of their host plant lineages (Kohler et al., 2015). Polyphyletic evolution of the ECM lifestyle is marked not only by convergent losses of different components of the ancestral saprotrophic apparatus but also by rapid genetic turnover in symbiosis-induced genes (Kohler et al., 2015). The ECM symbiosis is the most prominent mycorrhiza occurring in forest ecosystems. The tree supplies the ECM fungus with up to 30% of its photosynthesis-derived carbohydrates in return for up to 70% of its N and P needs, which are received from the ECM hyphal networks that extend deep within the soil (Nehls, 2008; Martin and Nehls, 2009). Thus, this mutualistic interaction relies on a constant nutrient exchanges between partners and contributes to better tree growth and health by improving mineral nutrition, strengthening plant defenses and directly contributing to the exclusion of competitive microbes (Wallander et al., 2001; Franklin et al., 2014).

Fungi release in the extracellular matrix a wide range of proteins to decay their substrates and to interact with their microbial, plant, or animal competitors and partners (Stergiopoulos and de Wit, 2009; Tian et al., 2009; Talbot et al., 2013; Essig et al., 2014). The fungal secretomes are composed of several protein categories, including proteases, lipases, Carbohydrate-Active enZymes (CAZymes), secreted proteins of unknown function and small-secreted proteins (SSP) (Alfaro et al., 2014). These secreted proteins either participate in organic matter degradation with hydrolytic enzymes such as CAZymes (Zhao et al., 2014), proteases or lipases or in interactions with their host through surface proteins like hydrophobins (Linder et al., 2005) or SSPs (Martin and Kamoun, 2011; van Ooij, 2011). ECM fungi have a reduced set of plant cell wall-degrading enzymes (Kohler et al., 2015). Over the past decade, SSPs have appeared as the cornerstone in the molecular dialog with host plants by altering host metabolism and/or defense responses in plant-microbe interactions (Kloppholz et al., 2011; Giraldo and Valent, 2013; Rovenich et al., 2014; Lo Presti et al., 2015). They were gradually associated with the term effector, which is defined as “microbial or pest secreted molecules that alter host-cell processes or structures and generally promote the microbe lifestyle” (Win et al., 2012). SSPs appear to play a key role in the ECM symbiosis (Plett and Martin, 2015). The protein MiSSP7 from *Laccaria bicolor* is required to establish symbiosis. This MiSSP is targeted to the host-plant nuclei where it interacts with the jasmonate co-receptor JAZ6 suppressing the plant defense reactions and allowing the development of the apoplastic Hartig net (Plett et al., 2011, 2014).

However, despite their ecological importance, little is known about the secretome of ECM fungi as most published analyses focused on the full genome repertoire of CAZymes, either secreted or not (Kohler et al., 2015). Only a few studies combined both *in silico* prediction of secretome and proteomic analysis of secretome (Vincent et al., 2012; Doré et al., 2015). To investigate whether the various components of the fungal secretome (CAZymes, proteases, lipases, SSPs) differ between ECM, pathogenic and saprotrophic species, we predicted, annotated and compared the secretomes of 49 fungal species, including 11 ECM symbionts recently sequenced (Kohler et al., 2015) and available via the JGI fungal genome portal MycoCosm (Grigoriev et al., 2014). Using this large set of predicted gene repertoires, we showed that the secretome size is not related to the fungal lifestyle. We also identified SSPs shared between ECM and saprophytic fungi, as well as lifestyle-specific SSPs.

## Results

### Correlation between secretome and fungal lifestyles

**Figure 1:**
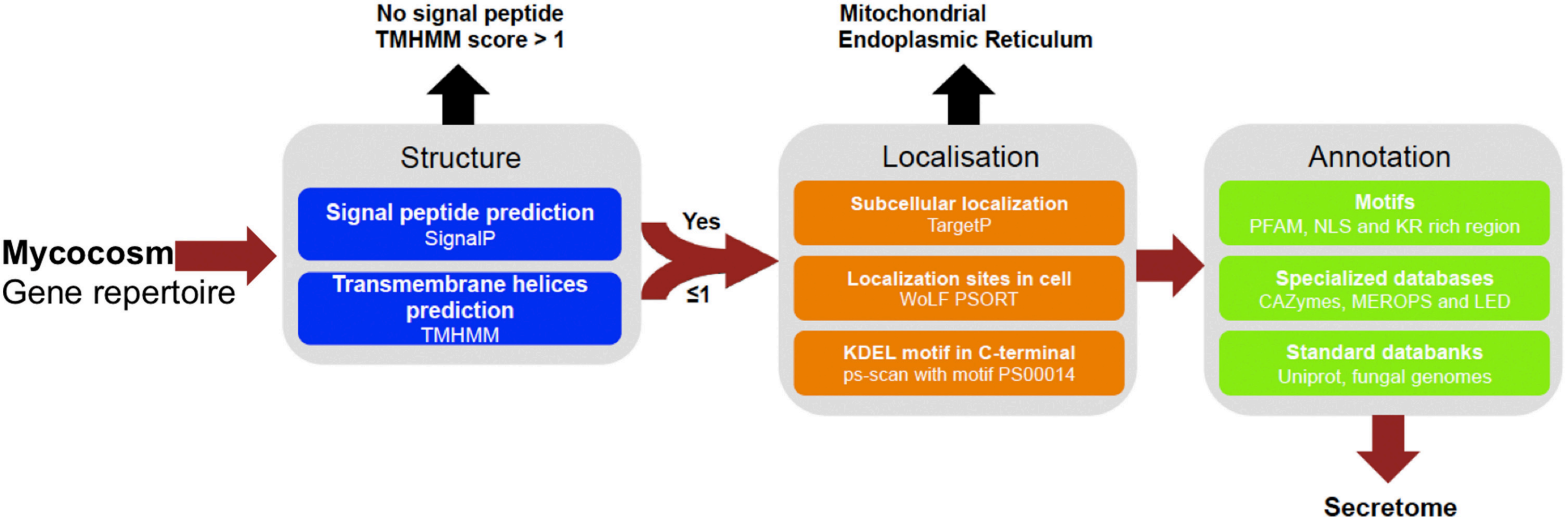
**Pipeline used to identify and annotate fungal secretome**. Secreted proteins have been predicted based on genomic sequences from 49 fungal genomes retrieved from Joint Genome Institute (JGI, url: http://genome.jgi.doe.gov/Mycorrhizal_fungi/Mycorrhizal_fungi.info.html). Prediction uses combined characteristics: proteins with signal-peptide as detected by SignalP v4.1 and no transmembrane domain or one overlapping signal peptide founded, no internal localization (no endoplasmic reticulum addressing “KDEL” motif, secretory pathway by TargetP v1.1 and extracellular by WoLF PSORT 0.2). Proteins have been annotated according to motives scanning (PFAM, NLS, KR rich region), homologies found in specialized databases (CAZy database, MEROPS for proteases and Lipase Engineering Databases) and standard databanks (Uniprot, Swissprot, Mycocosm). SignalP4.1 is set to ≪ sensitive mode ≫, the other softwares are set with default parameters.

**Figure 2:**
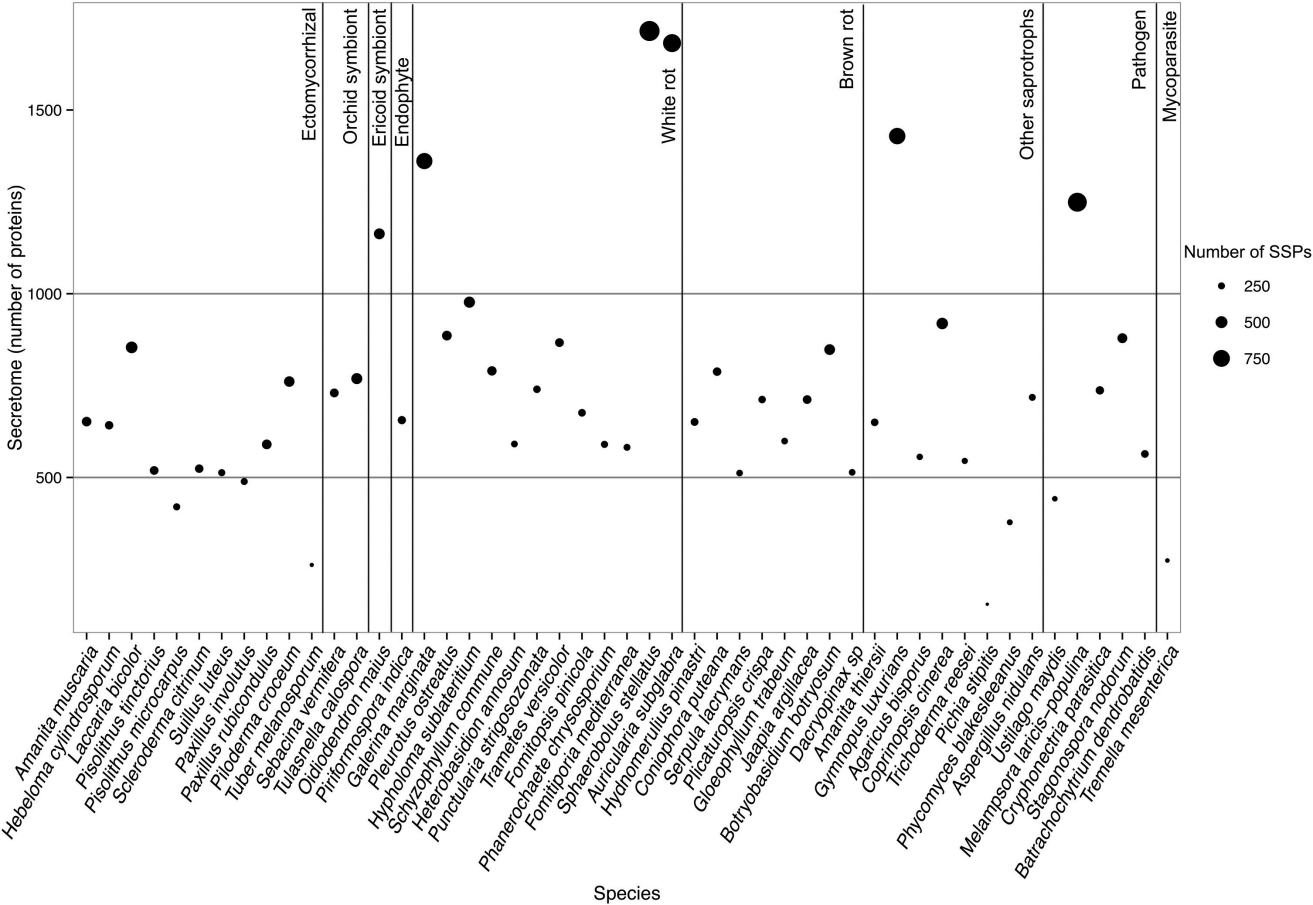
**Secretome size and richness in Small Secreted Proteins of each fungal species**. Species are ordered according to their lifestyles. Secretome size (number of proteins) is indicated on the y-axis. Size of the dot indicates richness in SSPs (number of proteins). Data obtained with *Spherobollus stellatus* and *Auricularia subglabra* have to be taken with caution due to poor annotation quality.

To determine whether secretomes and SSPs are related to biotrophic or saprotrophic lifestyles, we developed a pipeline to identify and compare the secretome from 49 fungal fungi including 41 Basidiomycota, six Ascomycota, one Zygomycota, and one Chytridiomycota (Figure 1, Table 1). Predicted secreted proteins contain an N-terminus type II secretion signal peptide, no transmembrane domain and do not contain sequences that retain them in organelles (mitochondria, plasts, ER, Golgi, etc.). Within the Basidiomycota, soil decayers and white-rot fungi display the largest secretomes. They displayed a wide range of sizes ranging from 155 to 1715 signal peptide-containing proteins (Figure 2). The total number of genes predicted in the fungal genomes analyzed ranged from 4000 to 25,000 and the proportion of secreted proteins (SP) from 3 to 10% of the total proteome. Most of the 49 secretomes analyzed (73.5%) contained between 500 and 1000 signal peptide (SP)-containing proteins (Figure 2). However, some secretomes are out of this range with a greater (12% of analyzed secretomes) or smaller (14 % of analyzed secretomes) number of secreted proteins. This excess or lower level of secreted proteins is not restricted to a single lifestyle, as three white-rot fungi (*Galerina marginata, Sphaerobolus stellatus*, and *Auricularia subglabra*), one litter decayer (*Gymnopus luxurians*) and one plant-biotrophic pathogen (*Melampsora larici populina)* possess more than 1000 SP-containing proteins (Figure 2). Plant pathogenic fungi have the largest proportion of secreted proteins. The size of the secretome from pathogenic- and white rot-fungi correlates with their proteome size, with a correlation coefficient *r*^2^ of 0.97 and 0.84, respectively (Supplementary Image 1A). In contrast, the secretome size from brown-rot decayers and ECM fungi does not correlate with the proteome size as they display a correlation coefficient of 0.57 and 0.30, respectively (Supplementary Image 1A). The fungi with the smallest secretomes are the ECM *Tuber melanosporum*, the mycoparasitic *Tremella mesenterica*, the litter decayer *Phycomyces blakesleeanus*, the plant biotrophic pathogen *Ustilago maydis* and the yeast *Pichia stipitis*.

**Table 1:** **List of fungal species analyzed in this study**.

**Name**	**Lifestyle**	**Phylum**	**Family**
*Agaricus bisporus*	Litter decayers	Basidiomycota	Agaricacae
*Amanita thiersii*	Litter decayers	Basidiomycota	Amanitacae
*Amanita muscaria*	Ectomycorrhizal	Basidiomycota	Amanitaceae
*Plicaturopsis crispa*	Brown rot	Basidiomycota	Amylocorticiaceae
*Piloderma croceum*	Ectomycorrhizal	Basidiomycota	Atheliales
*Auricularia subglabra*	White rot	Basidiomycota	Auriculariaceae
*Heterobasidion annosum*	White rot	Basidiomycota	Bondarzewiaceae
*Botryobasidium botryosum*	Brown rot	Basidiomycota	Botryobasidiaceae
*Coniophora puteana*	Brown rot	Basidiomycota	Coniophoraceae
*Punctularia strigosozonata*	White rot	Basidiomycota	Corticiaceae
*Hebeloma cylindrosporum*	Ectomycorrhizal	Basidiomycota	Cortinariaceae
*Dacryopinax sp*	Brown rot	Basidiomycota	Dacrymycetaceae
*Fomitopsis pinicola*	White rot	Basidiomycota	Fomitopsidaceae
*Sphaerobolus stellatus*	White rot	Basidiomycota	Geastraceae
*Gloeophyllum trabeum*	Brown rot	Basidiomycota	Gloeophyllaceae
*Fomitiporia mediterranea*	White rot	Basidiomycota	Hymenochaetaceae
*Jaapia argillacea*	Brown rot	Basidiomycota	Jaapiaceae
*Melampsora larici-populina*	Pathogen	Basidiomycota	Melampsoraceae
*Hydnomerulius pinastri*	Brown rot	Basidiomycota	Paxillaceae
*Paxillus involutus*	Ectomycorrhizal	Basidiomycota	Paxillaceae
*Paxillus rubicondulus*	Ectomycorrhizal	Basidiomycota	Paxillaceae
*Phanerochaete chrysosporium*	White rot	Basidiomycota	Phanerochaetaceae
*Pleurotus ostreatus*	White rot	Basidiomycota	Pleurotaceae
*Trametes versicolor*	White rot	Basidiomycota	Polyporaceae
*Coprinopsis cinerea*	Litter decayers	Basidiomycota	Psathyrellaceae
*Schizophyllum commune*	White rot	Basidiomycota	Schizophyllaceae
*Pisolithus tinctorius*	Ectomycorrhizal	Basidiomycota	Sclerodermataceae
*Pisolithus microcarpus*	Ectomycorrhizal	Basidiomycota	Sclerodermataceae
*Scleroderma citrinum*	Ectomycorrhizal	Basidiomycota	Sclerodermataceae
*Sebacina vermifera*	Orchid symbiont	Basidiomycota	Sebacinaceae
*Piriformospora indica*	Endophyte	Basidiomycota	Sebacinaceae
*Serpula lacrymans*	Brown rot	Basidiomycota	Serpulaceae
*Galerina marginata*	White rot	Basidiomycota	Strophariaceae
*Hypholoma sublateritium*	White rot	Basidiomycota	Strophariaceae
*Suillus luteus*	Ectomycorrhizal	Basidiomycota	Suillaceae
*Tremella mesenterica*	Mycoparasitic	Basidiomycota	Tremellaceae
*Laccaria bicolor*	Ectomycorrhizal	Basidiomycota	Tricholomataceae
*Gymnopus luxurians*	Litter decayers	Basidiomycota	Tricholomatocae
*Tulasnella calospora*	Orchid symbiont	Basidiomycota	Tulasnellales
*Ustilago maydis*	Pathogen	Basidiomycota	Ustilaginaceae
*Aspergillus nidulans*	Litter decayers	Ascomycota	Trichocomaceae
*Cryphonectria parasitica*	Pathogen	Ascomycota	Cryphonectriaceae
*Oidiodendron maius*	Ericoid symbiont	Ascomycota	Myxotrichaceae
*Pichia stipitis*	Yeast	Ascomycota	Saccharomycetaceae
*Stagonospora nodorum*	Pathogen	Ascomycota	Phaeosphaeriaceae
*Trichoderma reesei*	Litter decayers	Ascomycota	Hypocreaceae
*Tuber melanosporum*	Ectomycorrhizal	Ascomycota	Tuberaceae
*Batrachochytrium dendrobatidis*	Pathogen	Chytridiomycota	Incertae sedis
*Phycomyces blakesleeanus*	Litter decayers	Zygomycota	Phycomycetaceae

### Enrichment of functional categories

To identify ECM-specific proteins in secreted proteins with a known PFAM domain (if any), we performed an enrichment analysis on the protein repertoires from species belonging to five fungal lifestyles (white-rot and brown-rot decayers, litter decayers, ECM, and plant pathogens; Table 2). Most of the PFAM domains identified in secreted proteins are related to enzymatic activities [proteases, lipases, and glycosyl hydrolases (GH)]. None of the enriched categories of PFAM-containing secreted proteins is shared by all ECM fungi studied (Table 2).

**Table 2:** **PFAM enrichment analysis in fungal secretomes**.

**Lifestyles**	**PFAM**	**Description**
Plant pathogen	PF07732	Multicopper oxidase
	PF07731	Multicopper oxidase
	PF00082	Peptidase_S8
	PF00394	Multicopper oxidase
	PF00026	Eukaryotic aspartyl protease
	PF00150	Glycoside hydrolase family 5
	PF00135	Carboxylesterase
	PF01565	FAD binding domain
	PF01083	Cutinase
	PF00722	Glycosyl hydrolases family 16
Brown rot	PF09286	Pro-kumamolisin
	PF00135	Carboxylesterase
	PF13668	Ferritin-like
	PF00722	Glycosyl hydrolases family 16
	PF00082	Peptidase_S8
	PF00026	Eukaryotic aspartyl protease
	PF00150	Glycoside hydrolase family 5
	PF00657	GDSL-like Lipase/Acylhydrolase
	PF01764	Lipase (class 3)
	PF01915	Glycosyl hydrolase family 3 C-terminal domain
	PF00704	Glycosyl hydrolases family 18
	PF00933	Glycosyl hydrolase family 3 N terminal domain
	PF00450	Peptidase_S10
	PF03330	RlpA like double-psi beta-barrel
White rot	PF09286	Pro-kumamolisin
	PF00135	Carboxylesterase family
	PF00722	Glycosyl hydrolases family 16
	PF00082	Peptidase S8
	PF00026	Peptidase S8
	PF00150	Glycoside hydrolase family 5
	PF01764	Lipase (class 3)
	PF03443	Glycoside hydrolase family 61
	PF01185	Hydrophobin
	PF00450	Serine carboxypeptidase
	PF03330	RlpA like double-psi beta-barrel
	PF10342	Ser-Thr- rich GPI-anchored membrane protein
Litter decayers	PF07732	Multicopper oxidase
	PF00082	Peptidase S8
	PF00026	Eukaryotic aspartyl protease
	PF00704	Glycosyl hydrolases family 18
Ectomycorrhizal	none	

PFAM enrichment analysis in secreted proteins compared to non-secreted proteins has been performed according to Saunders et al. (2012). Enriched PFAM domains shared by all members of one fungal lifestyle (plant pathogen, white rot, brown rot, litter decayers, and ectomycorrhizal) are shown.

### Composition of fungal secretomes by CAZymes, proteases, and lipases

**Figure 3:**
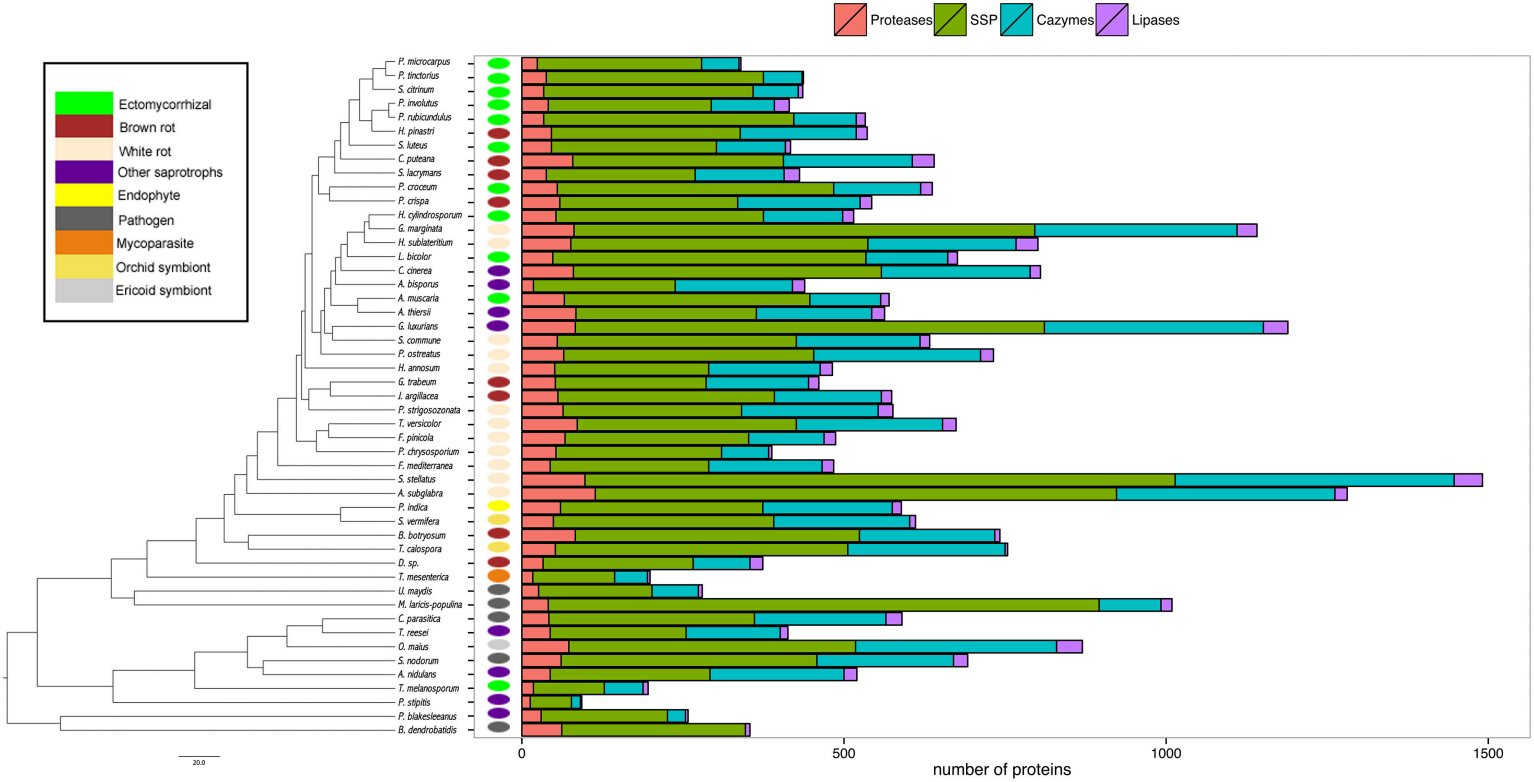
**Global composition of 49 fungal secretomes**. Predicted secretomes have been annotated based on four functional categories (Proteases, SSP, CAZymes, Lipases). Sizes of secretomes are presented as absolute number. Bars represent number of proteins in each category of secreted proteins. Phylogenetic tree is adapted from Kohler et al. (2015).

Our analysis then focused on four categories (i.e., proteases, lipases, CAZymes, and SSPs) known for their biological and ecological relevance in saprotrophic and ECM fungi. In this study, we defined SSPs as predicted secreted proteins smaller than 300 amino acids. Secretomes have been annotated according to similarities with available databases or a chosen cut-off (Figure 3). SSPs are the most abundant secreted proteins in all species analyzed, followed by CAZymes, proteases and finally lipases (Figure 3).

#### CAZymes

The average number of secreted PCW-degrading CAZymes of ECM fungi is significantly reduced when compared to the one of the other fungal lifestyles, except plant pathogens (Supplementary Image 2, Supplementary Data sheet 1). A detailed analysis of the CAZyme families and their evolution is provided in Kohler et al. (2015).

#### Proteases

**Figure 4:**
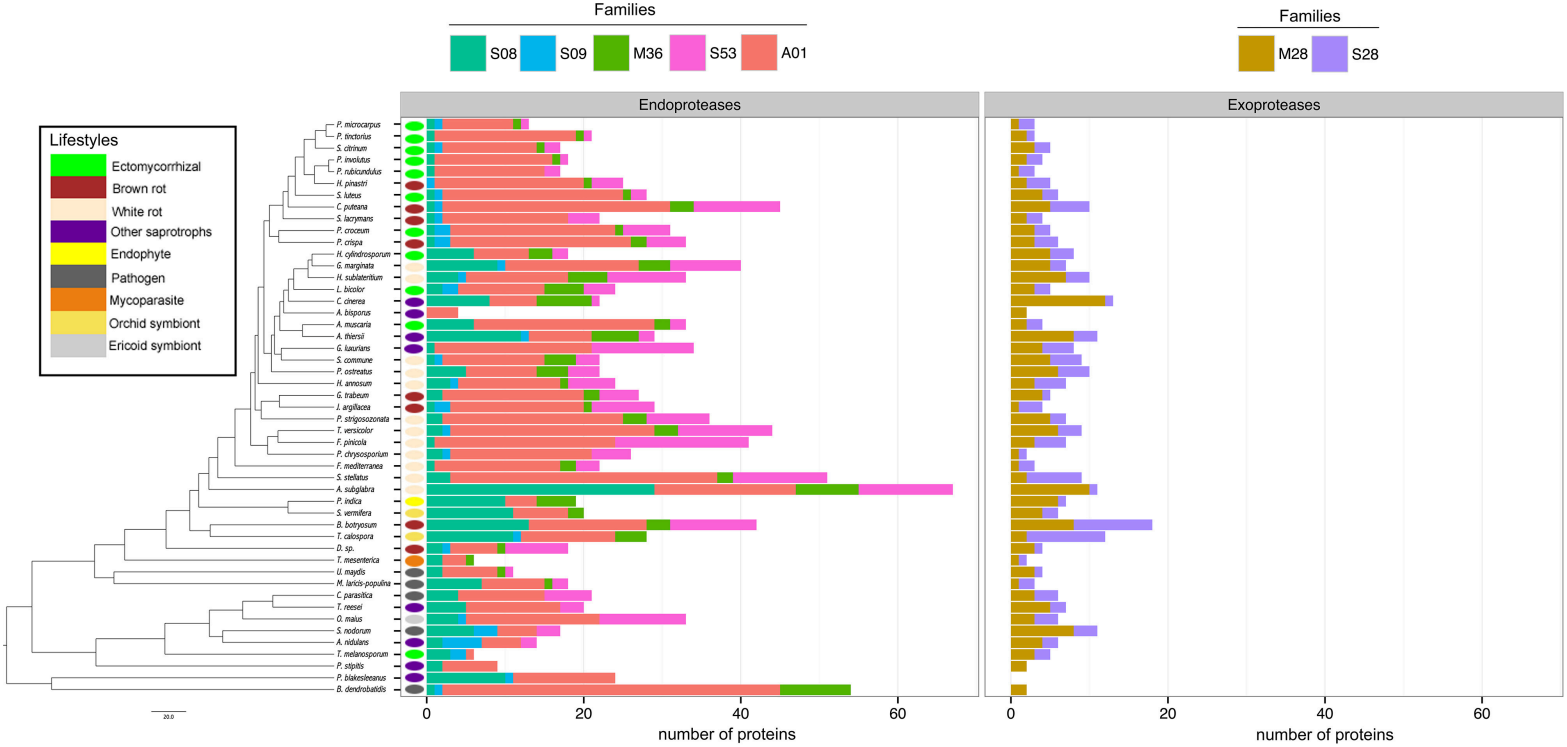
**Fungal secretome's composition in exoproteases and endoproteases**. A BLASTP (Altschul et al., 1990) search against MEROPS database was performed with all secreted protein sequences. The secreted proteins were subdivided in two main categories: exoproteases and endoproteases. Endoproteases: A01 (Pepsin A family); M36 (Fungalysin family); S53 (Sedolisin family); S28 (lysosomal Pro-Xaa carboxypeptidase family). Exoproteases: S08 (Subtilisin family); S09 (carboxypeptidase family); M28 (aminopeptidase family). Phylogenetic tree is adapted from Kohler et al. (2015).

To assess the protease capability of the analyzed fungi (mainly found in forest ecosystems), we performed a BLASTP search against the MEROPS database. We considered families S08, S09, M36, S53, and A01 for endoproteases and families M28 and S28 for exoproteases as they are ecologically relevant for ECM (Rineau et al., 2013). We then subdivided the proteases into exoproteases and endoproteases. Proteases represent approximately 7% of the secretome, ranging from 6 endo- or exoproteases for the litter decayer *Agaricus bisporus* to 78 proteases for the white-rot fungus *A. subglabra*. Both types of proteases are present in all studied species (Figure 4). Endoproteases are present in higher numbers than exoproteases independently of the lifestyle (Figure 4). No significant differences were found between biotrophic and saprotrophic species (Supplementary Image 3), indicating that ECM fungi have conserved the protease ability of their saprotrophic cousins.

#### Lipases

**Figure 5:**
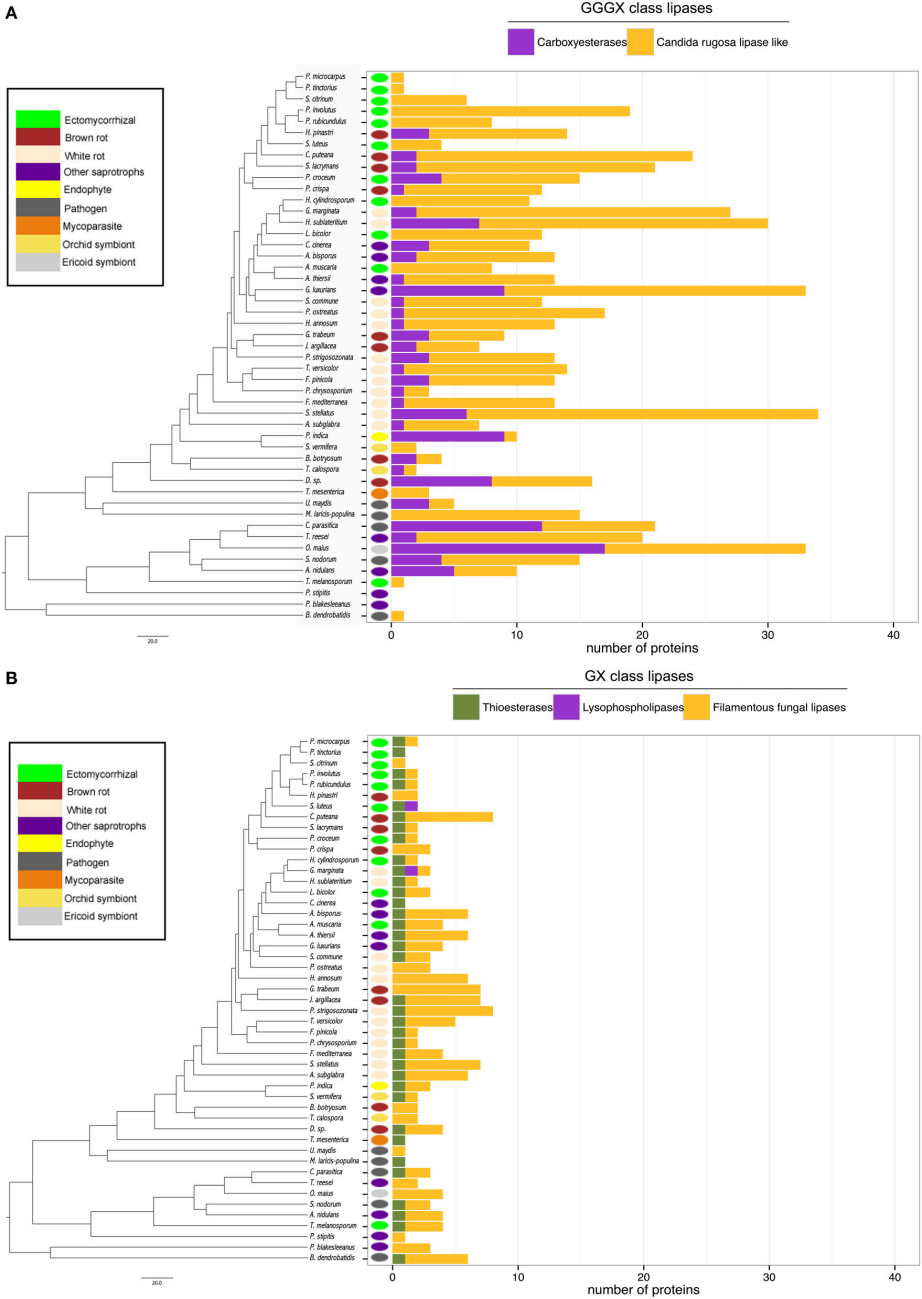
**Lipasic ability of fungal secretome**. BLASTP searches against Lipase Engineering Databases were performed using the full set of secreted proteins. Sequences were thus divided in two classes: GGGX class **(A)** and GX class **(B)**. Phylogenetic tree is adapted from Kohler et al. (2015).

Lipases are the less represented secreted proteins, regardless of the fungal lifestyles. The secretome of the white-rot fungus *S. stellatus* contains the highest number of secreted lipases (44), whereas the ECM symbiont *Pisolithus tinctorius* and the yeast *Pichia stipitis* have only two secreted lipases. Genome-wide analysis of secreted lipases shows differences between GX and GGGX lipase classes (Figure 5), the two main classes used by the Lipase Engineering Database to classify lipases. Among the 49 fungal secretomes, the GGGX family, which contains carboxylesterases and *Candida rugosa* lipase-like (CRL), is more represented than the GX class (Figure 5). Two fungi (the white rot *Phycomyces blakesleeanus* and the yeast *Pichia stipitis*) have no GGGX class lipases (Figure 5A). ECM fungi lack secreted carboxylesterases (CE) from the GGGX family, except for *Piloderma croceum* (Figure 5A), contrasting with the ericoid symbiont *Oidiodendron maius* presenting a high number of both CE and CRL. Among GX class lipases, only two fungi (the ECM symbiont *Suillus luteus* and the white rot *G. marginata*) have lysophospholipases (Figure 5B). By contrast, thioesterases are found among all ECM fungi except *Scleroderma citrinum* (Figure 5B). ECM fungi display a low repertoire of filamentous fungal lipases compared with white rot fungi (Supplementary Image 4).

### Presence of SSPs in ECM secretomes

**Figure 6:**
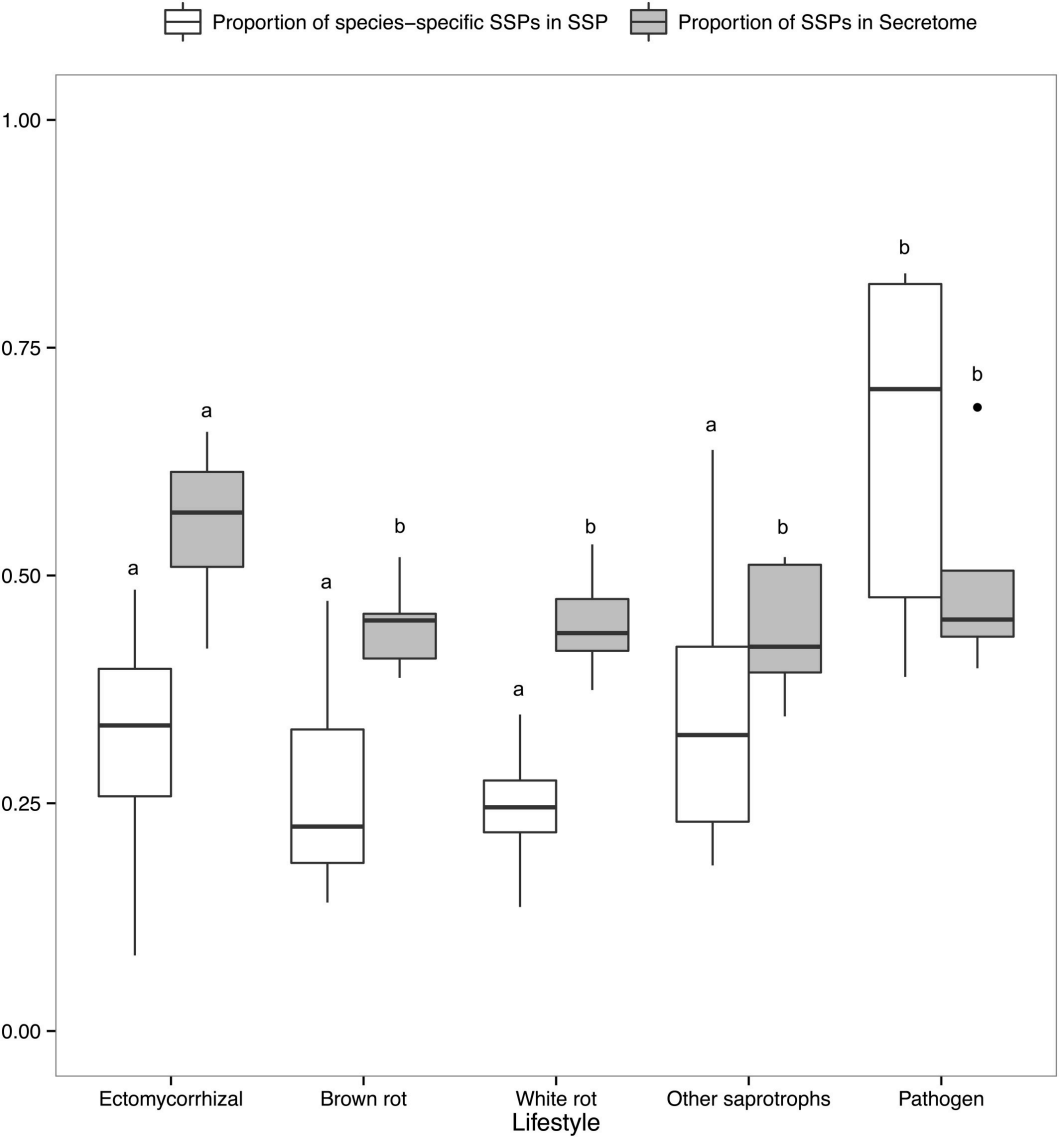
**Proportion of SSPs and species-specific SSPs**. Boxplots showing proportion of SSP in secretome across lifestyles (gray) and proportion of species-specific SSPs, defined as SSPs with no homology in other species (white). Different letters shows significant difference (*p* < 0.01) based on pairwise *t*-test with holm correction of *p*-values. Only fungal lifestyles containing at least five fungal species have been taken in account.

There is a positive correlation between the number of SSPs and the secretome size, with the largest secretomes having the highest number of SSPs (Supplementary Image 1B). ECM fungi display significantly higher percentage of SSPs in their secretome than saprophytic species (Figure 6), whereas this percentage does not significantly vary between the other lifestyles. Species-specific SSPs (no sequence similarity within the set of compared fungi using BLASTP) are more abundant in plant pathogens, whereas they display similar levels in other lifestyles (Figure 6). Overall, ECM fungi are enriched in SSPs compared to the other fungal lifestyles, but do not display significantly higher numbers of species-specific SSPs.

### Identification of shared- and lifestyle-specific SSPs

**Figure 7:**
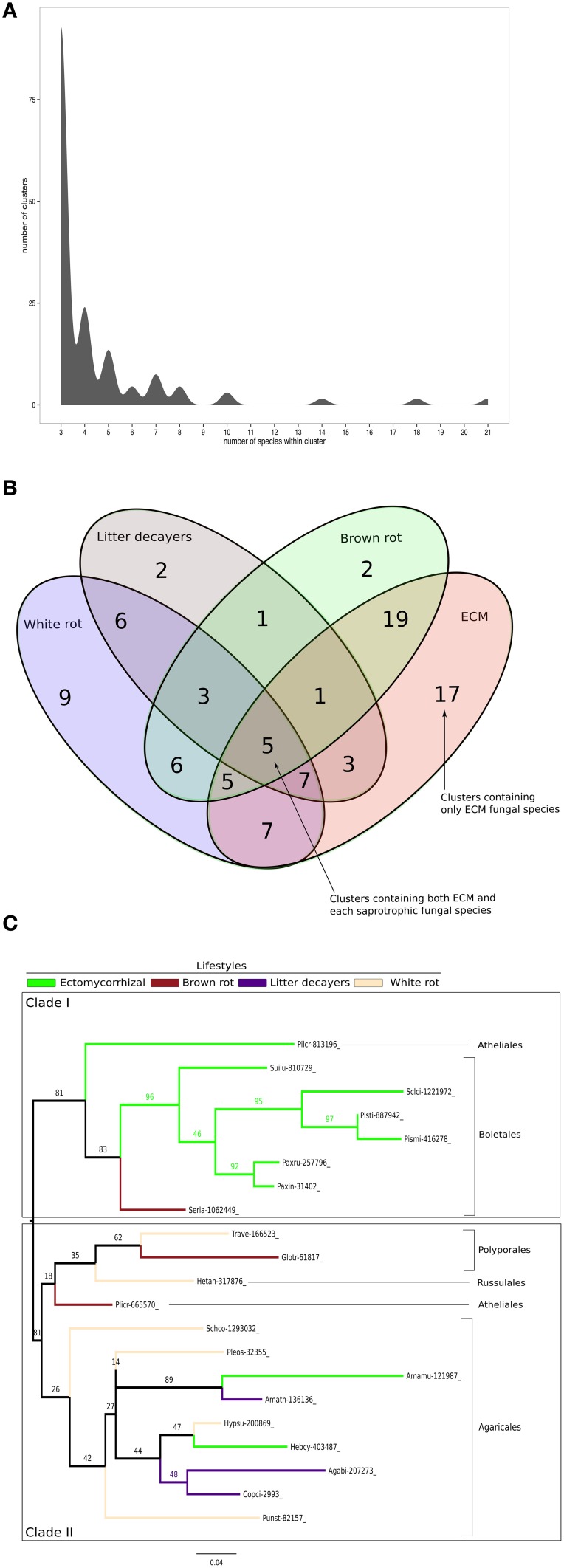
**Shared Small-Secreted Proteins among four different lifestyles. (A)** Number of clusters involving at least three different species. **(B)** Four sets Venn diagram showing number of clusters of SSPs shared between 11 ECM fungi, 2 orchid symbionts, 12 white rot, 8 brown rot, and 7 soil or litter decayer fungi, based on CD-HIT clustering with identity threshold set to 70%. **(C)** Phylogenetic tree inferred from 1000 bootstraps using maximum likelihood phylogeny of the largest cluster containing SSPs from both ECM fungi and each of saprotrophic fungal species (white rot, brown rot, litter decayer). Bootstrap values are shown at the corresponding node. Colors indicate fungal lifestyle and orders of the different fungi are given.

To identify conserved SSPs shared between the 28 saprotrophic (white rot, brown rot, and soil and litter decayers) and 14 mycorrhizal fungi (orchid, ericoid and ECM symbionts), we performed a clustering analysis based on sequence identity using CD-HIT software with an identity threshold set to 70% (Figure 7). The clustering analysis has been performed on a total of 16,821 SSP sequences and generated a total of 14,284 clusters, of which 101 clusters contain SSPs from at least three different fungal species (Figure 7A). Only the latter clusters have been kept for further analysis. Most of the defined clusters are species-specific (clusters with only one species) (Supplementary Data sheet 2). There is no cluster containing SSPs shared between all lifestyles, but seven SSP clusters are shared between ECM and saprotrophic fungi only (Figure 7B). PFAM annotations indicate that those proteins are related to fungal proteins of unknown function and uncharacterized domains (PF09435 and PF08520), thaumatin (PF0314), GH 25 (PF01183), cyclophylin (PF00160), and ADP-ribosylation family (PF00025) (Table 3).

**Table 3:** **Small-secreted proteins shared among lifestyles**.

**Cluster id**	**Nb. of species inside clusters**	**Protein length (min–max)**	**PFAM domain**	**Fungal species within clusters**	**Description**
**CLUSTERS CONTAINING ONLY ECM FUNGAL SPECIES**					
92	5	234–236	None	*P. involutus, P. rubicondulus, P. microcarpus, P. tinctorius, S. citrinum*	No PFAM domain
97	5	194–206	PF02845	*P. involutus, P. rubicondulus, P. microcarpus, P. tinctorius, S. citrinum*	CUE domain
52	3	110–257	PF01183	*P. involutus, P. rubicondulus, Suillus luteus*	Glycosyl hydrolases family 25
154	4	220	PF13460	*P. involutus, P. rubicondulus, S. citrinum, S. luteus*	NADH(P)-binding
169	4	132–189	PF10342	*P. involutus, P. rubicondulus, P. microcarpus, P. tinctorius*	Ser-Thr-rich gpi-anchored family
126	3	293–294	PF01764	*P. microcarpus, P. tinctorius, S. citrinum*	Lipase (class 3)
143	3	98–246	PF10342	*P. involutus, P. rubicondulus, S. luteus*	Ser-Thr-rich gpi-anchored family
147	3	160–234	PF04777	*P. microcarpus, P. tinctorius, S. citrinum*	Erv1 / Alr family
251	3	152–263	None	*P. microcarpus, P. tinctorius, S. citrinum*	No PFAM domain
264	3	241–252	None	*P. microcarpus, P. tinctorius, S. citrinum*	No PFAM domain
267	3	208–249	PF00491	*P. involutus, P. rubicondulus, P. microcarpus*	Arginase family
292	3	225–229	PF03227	*P. microcarpus, P. tinctorius, S. citrinum*	GILT/Thiol oxidoreductases
373	3	148–153	None	*P. microcarpus, P. tinctorius, S. citrinum*	No PFAM domain
381	3	141–145	PF07249	*P. microcarpus, P. tinctorius, S. luteus*	Cerato-platanin
385	3	144	PF07249	*P. microcarpus, P. tinctorius, S. citrinum*	Cerato-platanin
429	3	113	None	*P. microcarpus, P. tinctorius, S. citrinum*	No PFAM domain
473	3	55–77	None	*P. involutus, P. microcarpus, S. citrinum*	No PFAM domain
**CLUSTERS CONTAINING BOTH ECM AND SAPROTROPHIC FUNGAL SPECIES**					
0	21	179–186	PF00025 PF00071	*A. bisporus, A. muscara, A. thiersii, C. cinerea, G;trabeum, H. cylindrosporum, H. annosum, H. sublateritium, P. involutus, P. rubicondulus, P. croceum, P. microcarpus, P. tinctorius, P. ostreatus, P. crispa, P. strigozonata, S. commune, S. citrinum, S. lacrymans, S. luteus, T. versicolor*	ADP-ribosylation family Ras family
1	18	217–240	PF00160	*A. bisporus, A. muscara, A. thiersii, F. pinicola, G. trabeum, G. luxurians, H. annosum, H. sublateritium, L. bicolor, P. rubicondulus, P. chrysosporium, P. microcarpus, P. tinctorius, P. crispa, P. strigosozonata, S. citrinum, S. lacrymans, T. versicolor*	Cyclophylin
12	10	224–247	PF01183	*A. bisporus, A. subglabra, G. trabeum, P. rubicondulus, P. chrisosporium, P. ostreatus, P. strigosozonata, S. commune, S. lacrymans, T. versicolor*	Glycosyl Hydrolase 25
25	8	67–73	PF08520	*A. thiersii, F. pinicola, H. annosum, J. argillacea, L. bicolor, P. crispa, S. luteus, T. versicolor*	Fungal protein of unknown function
50	5	254–257	PF00314	*C. cinerea, F. mediterranea, H. cylindrosporum, H. sublateriutium, J. argillacea*	Thaumatin family
**CLUSTERS CONTAINING ONLY SAPROTROPHIC FUNGAL SPECIES (WHITE ROT, BROWN ROT, LITTER DECAYERS)**					
16	7	238–257	PF03443	*G. luxurians, H. annosum, J. argillacea, P. ostreatus, P. crispa, P. strigosozonata, T. versicolor*	Glycosyl Hydrolase 61
56	5	242–248	PF03443	*A. bisporus, A. thiersii, P. crispa, S. lacrymans, T. versicolor*	Glycosyl Hydrolase 61
217	3	105–110	PF01185	*F. mediterranea, G. luxurians, J. argillacea*	Fungal hydrophobin

List of clusters containing only small-secreted proteins from ectomycorrhizal fungal species (ECM), both ectomycorrhizal and saprotrophic fungal species and only saprotrophic fungal species (white rot, brown rot, litter decayers). PFAM analysis were performed with MotifFinder with an e-value cut off of 1.10^-08^, based on CD-HIT clustering analysis with identity threshold set to 70%.

ECM fungi share most of their SSPs with brown rot (19 clusters), white rot (7 clusters), and litter decayers (3 clusters) (Figure 7B). Moreover, 17 clusters are specific to ECM fungi (Table 3). These clusters contain proteins with PFAM domains associated with ceratoplatanin (PF07249), Ser-Thr-rich glycosyl-phosphatidyl-inositol-anchored membrane proteins (PF10342), GH 25 (PF01183), CUE domains likely binding ubiquitin (PF02845), NADPH-binding protein (PF13460), thiol-oxydoreductases (PF07249), and Evr1/Alr family proteins likely involved in the maturation of Fe/S clusters (PF04777). Six clusters specific for ECM fungi contain SSPs without any PFAM domain, suggesting the presence of lifestyle-specific SSPs among ECM symbionts. Finally, SSPs specific to saprotrophic fungi include fungal hydrophobin (PF01185) and GH 61 (PF03443) sequences. To harness evolution of SSP-encoding genes, we performed phylogenetic analysis on the cluster containing the highest number of SSPs from saprotrophic (white rot, brown rot, and litter decayer) and ectomycorrhizal fungi (Figure 7C). Two clades are highly supported by bootstraps. Clade I contains mainly Boletales and one Atheliales, whereas Clade II contains mainly Agaricales and Polyporales. These two clades are highly consistent with taxonomy and are thus not independent from it. Most interestingly, clade I is enriched but not exclusively with ECM fungi, whereas clade II is enriched but not exclusively with saprotrophic fungi. This analysis is supporting an independent diversification of SSPs. However, clade I contains among seven ECM fungi *Serpula lacrymans*, a brown-rot fungus and clade II contains among 11 saprotrophic fungi, two ECM fungi: *Hebeloma cylindrosporum* and *Amanita muscaria*. This suggests that SSPs of ECM fungi may have evolved from their saprotrophic ancestors.

## Discussion

This genomic comparative genomic study is based on *in silico* analysis and bioinformatics tools. The results should thus be taken with care as they may be impacted by both qualities of sequencing (Supplementary Data sheet 3) and annotation tools used. For instance, secretome size found for two white rot fungi, *S. stellatus* and *A. subglabra*, are strongly higher than any other fungal secretome analyzed in this study (1715 and 1682 predicted secreted proteins, respectively). Moreover, proteins can be secreted using unconventional secretion system (Nickel and Rabouille, 2009) and as they do not contain any signal peptide, they are not predicted as secreted proteins using our bioinformatics pipeline. For instance, proteomic analysis of *H. cylindrosporum*'s exoproteome identified 228 secreted proteins not computationally predicted as secreted (Doré et al., 2015). On other hand, a proteomic analysis on *L. bicolor* free-living mycelium revealed 815 secreted proteins using SDS-PAGE shotgun method (Vincent et al., 2012), a number similar to the one of *in silico* predicted secreted proteins (854 proteins predicted as secreted). However, the two exoproteome are different in their composition (Vincent et al., 2012). Vincent et al., also highlighted that the experimental approach used for separation and identification of proteins has an impact on the detection of secreted proteins (Vincent et al., 2012). Therefore, combination of both computational and experimental approaches appears as the most accurate strategy to study fungal secretome's composition.

### Fungal secretomes, not a matter of size

In this study, we predicted, analyzed and compared the secretome of 49 fungal species, including 11 ECM fungi. We focused our analysis on ECM secretomes aiming to identify shared- or lifestyle-specific features. A previous analysis on 33 microbial secretomes (mostly Ascomycota and Oomycota) showed that the number of predicted secreted proteins was related to the phylogenetic relationships between species rather than their ecological traits (i.e., lifestyles) (Krijger et al., 2014). Our study, mostly performed on Basidiomycota, confirms and extends those conclusions by showing that most of the analyzed secretomes contain 500–1000 proteins and their size is not related to the species lifestyle. However, a recent proteomics survey of secreted proteins involved in lignocellulose degradation in Basidiomycota highlighted differences between fungi having different lifestyles. The authors suggested that the lifestyle shapes the composition, but not the size of fungal secretomes (Alfaro et al., 2014). In the present study, we surveyed the secretome composition of ECM fungi and compared it to saprotrophic and pathogenic species.

### ECM fungi have a reduced repertoire of secreted PCW-degrading CAZymes

CAZymes are important for both organic matter degradation and colonizing the host in pathogen/symbiotic interactions by facilitating the breakdown of plant cell wall components (van den Brink and de Vries, 2011; Zerillo et al., 2013; Brouwer et al., 2014). ECM fungal genomes have lost genes encoding for CAZymes and lytic polysaccharide mono-oxygenases (Kohler et al., 2015), similar to what has been observed in brown rot fungi, and likely due to their intercellular interactions with plants (Eastwood et al., 2011; Floudas et al., 2012). Our data detail these results by showing that ECM fungi secrete less PCW active CAZymes than white rot fungi, although we did not find any statistically significant differences when we compared ECM fungi with brown rot fungi or other saprotrophs (i.e., soil and litter decayers or yeast). The remaining plant cell wall degradative capacities are likely involved both in the presymbiotic phase, where ECM fungi are not yet in symbiosis with a host, and during the first steps of the colonization process, where the ECM fungus needs to penetrate the plant root cortex.

### ECM and saprotrophic fungi have similar protease and lipase repertoires

In most temperate and boreal forest soils, organic matter is predominant. The role of ECM fungi in soil organic matter degradation and/or modification is known and required decomposition abilities. However, the consequence of soil organic matter modification by ECM fungi in particular for carbon storage is under debate (Lindahl and Tunlid, 2015). Nitrogen is mainly found in an organic form, such as proteins, and carbon in complex carbohydrates. Recent studies using the ECM fungus *Paxillus involutus* suggest that glucose (corresponding to carbon provided by the plant) controls the assimilation of organic nitrogen by this ECM fungus (Rineau et al., 2012; Shah et al., 2013). Mobilization of nitrogen in organic matter requires the action of secreted proteases (Geisseler et al., 2011). Another recent study proposes that endoproteases from the A01, M36, and S53 families in combination with exoproteases (M28, S28, and S9 families) are likely required to degrade soil proteins (Shah et al., 2013). Additionally, subtilisins (S08 family) are dominant in the secretomes of saprotrophic fungi (Hu and Leger, 2004). Most mycoparasitic and pathogenic fungi display an elevated number of subtilisin-like serine proteases (Muszewska et al., 2011) that play a key role in these interactions (Bryant et al., 2009). In contrast, ECM fungi display fewer subtilisins (S08 family) in comparison with white rot fungi, endophytes, ericoid fungi, and pathogenic fungi. This reduced set of subtilisins appears specific to the ECM fungi (and to a lesser extent to several brown rot fungi taxonomically-related to ECM fungi). The reduced number of subtilisins might be a way for ECM fungi to avoid eliciting plant defense mechanisms (Figueiredo et al., 2014).

In plant pathogenic fungi, secreted lipases are involved in propagule adhesion and plant tissue penetration to promote colonization (Voigt et al., 2005; Chu et al., 2008). They may also be used to facilitate nutrient absorption from the host or involved in the inhibition of immunity-related callose formation (Blümke et al., 2014). In *Magnaporthe oryzae*, a lipase-like protein up-regulated during plant penetration and biotrophic development is likely involved in both appressorium and manipulation of cell to cell communication through plasmodesmata (Oliveira-Garcia E; 28th Fungal Genetics Conference, Pacific-Groove, CA, USA). However, in mutualistic interactions, no functional studies have described the role of secreted lipases. Because levels of secreted lipases is low in each fungal lifestyle, it is not surprising that we do not see a reduction in secreted lipases similar to what has been observed with secreted CAZymes.

### ECM fungi share SSPs with saprotrophic fungi, but also display symbiosis-specific SSPs

SSPs have been extensively studied in recent years for their involvement in host-pathogen interactions as effectors (Giraldo and Valent, 2013). However, those SSPs have been found in most fungal species regardless of their lifestyle. Interestingly, several SSPs are secreted by free-living mycelium and are thus non-specific to symbiotic tissues (Vincent et al., 2012; Doré et al., 2015). This suggests a role of SSPs in the biology of extramatrical mycelium, which is the one interacting with rhizospheric microbes. These secreted proteins are likely involved in a variety of processes including differentiation of fungal structure, such as fruiting body, (e.g., hydrophobins), cell-to-cell communication (Murphy et al., 2012), competition between fungi (Trejo-Hernández et al., 2014) and fungal-host interactions (Chisholm et al., 2006; Plett and Martin, 2015). Our genome-wide survey reflects the versatility of SSPs with both shared- and lifestyle-specific proteins. Among the 17 SSP clusters only found in ECM fungi, we found many proteins identified as ceratoplatanins. Ceratoplatanin is a fungal elicitor of plant defenses and is therefore considered to be involved in pathogen-associated molecular patterns (de Oliveira et al., 2011; Baccelli et al., 2014). Finding ECM-specific ceratoplatanins in mutualistic fungi suggested that that they are involved in microbial-associated molecular patterns (MAMP) involved in polysaccharide recognition. Additionally, a recent study showed that the ceratoplatanin ELP1 from the soil fungus *Trichoderma atroviride* forms highly ordered monolayers at a hydrophobic surface/liquid-interface and hybrid ordered layers when added to hydrophobins (Bonazza et al., 2015).

Several of the PFAM domains found in ECM-specific sequences, such as Ser-Thr/GPI rich anchored proteins and the lysozyme-like GH 25, are also related to cell wall remodeling and/or organization. Interestingly, these PFAM domains are enriched in the secretomes of rust fungi (Saunders et al., 2012), suggesting a possible role for the biotrophic way of life. Ser-Thr-GPI anchored proteins may be involved in the development of fruiting bodies (Frey et al., 2015) as well as in signaling when they interact with MAPK (Shen et al., 2015). Six clusters do not have any known PFAM domains, suggesting new families of ECM-specific SSPs. Finally, three SSP clusters are specific to saprotrophic fungi; they include fungal hydrophobin (PF01185) and copper-dependent lytic polysaccharide monooxygenases GH 61/AA9 (PF03443) (Levasseur et al., 2013). Orchid mycorrhizal symbionts do not share SSP clusters with ECM fungi. However, they share three SSP clusters with white rot fungi and a single SSP cluster with both white and brown rot fungi. This suggests that orchid symbionts are more similar to saprotrophic fungi and is consistent with a recent comparative genomic study (Martino, personal communication). Overall, ECM fungi share a large set of SSPs with brown rot fungi, white rot fungi and litter decayers in that order, supporting the view of a continuum between ECM fungi, rot fungi and litter-decayer fungi (Riley et al., 2014). Phylogenetic analysis confirms at least some SSPs from ectomycorrhizal fungi have evolved from SSPs found in saprotrophic ancestors. Knowing that MiSSP7 from *L. bicolor* is an effector protein required for ECM symbiosis establishment (Plett et al., 2014), one could wonder whether this SSP evolved from saprotrophic fungi. We finally identified a number of SSPs specific to the ECM fungi, which make them good candidates for further functional analyses.

Altogether, those results support the concept of a continuum from saprophytic to ECM fungi, where ECM fungi share common secreted proteins with their saprophytic cousins, but also contain ectomycorrhiza-specific SSPs likely involved in the fine-tuning of the mutualistic interaction established with their host(s). The present findings confirmed and extended the large scale genome analysis showing that emergence of ECM symbiosis has been associated with a loss of plant cell wall degrading enzymes and a rapid turn-over of symbiosis-related genes (Kohler et al., 2015). Overall, SSPs represent a significant part of the fungal secretomes analyzed, especially among ECM fungi. The fact that ECM fungi are enriched in SSPs compared with the other lifestyles might reflect the conservation of SSPs from saprotrophic ancestors and the expansion of symbiosis-specific SSPs dedicated to the molecular cross-talk between partners, the accommodation of hyphae *in planta*, the establishment and functioning of the symbiosis (Garcia et al., 2015). This stresses the need for functional analysis of the candidate effectors to allow for more accuracy in future computational studies (Sperschneider et al., 2015).

## Methods

### Bioinformatics pipeline and functional annotation of fungal secretomes

Prediction of secreted proteins was performed using a custom bioinformatic pipeline (Figure 1) assessing the following combined sequence characteristics: (a) proteins were predicted as secreted if the presence of a signal peptide was detected with SignalP, with D-cutoff values set to “sensitive” (version 4.1; option eukaryotic; Petersen et al., 2011), and no transmembrane helix or one overlapping the signal peptide found by TMHMM using default parameters (version 2.0; Melén et al., 2003) and (b) protein subcellular localization. Proteins were considered as secreted if subcellular localization was assigned as a secretory pathway using TargetP with the –N option to exclude plants (version 1.1; Emanuelsson et al., 2000) and as extracellular with WolfPsort using the option “fungi” (version 0.2; Horton et al., 2007). To filter out proteins that permanently reside in the endoplasmic reticulum (ER) lumen, we scanned the proteins for the KDEL motif (Lys-Asp-Glu-Leu) in the C-terminal region (prosite accession “PS00014”) with PS-SCAN (version 1.79). Annotation of the secreted proteins was completed by a BLASTP query comparing protein sequences against different resources and specialized databases (*e*^value^ = 10^−5^ and choosing the best hit) using the followingdatabases: (1) CAZyme (http://www.cazy.org/), (2) MEROPS (http://merops.sanger.ac.uk/), and (3) Lipase Engineering Database (http://www.led.uni-stuttgart.de/) and the following international DNA databases: (1) Uniprot Swissprot and (2) JGI Mycocosm. We also performed domain searches with the HMMER package (version 3.0, default parameters; Finn et al., 2011) for PFAM domains. To predict whether the secreted proteins targeted nuclei, we used PredictNLS (default parameters, version 1.0.20; https://rostlab.org/owiki/index.php/PredictNLS) for determine the presence of a nuclear localization signal. We also estimated the percentage of cysteine and the KR-rich regions of the secreted proteins. We considered secretome proteins smaller than 300 amino acids as SSPs. Data mining and comparison and figure plotting have been performed using the R software (R Core Team, 2014, http://www.R-project.org/) and an *in-house* Python script.

### Clustering analysis and venn diagram

The clustering analysis has been performed based on sequence identity with CD-HIT software (Huang et al., 2010). The identity threshold has been set to 70% and only clusters involving at least three different fungal species have been taken in account. Protein sequences have been retrieved with an *in-house* Python script. Clustering analysis data have been used to generate a 4-sets Venn diagram.

### Phylogenetical and statistical analysis

Phylogenetic analysis of the largest cluster containing both saprotrophic and ectomycorrhizal fungal species has been performed on protein sequences using MAFFT v7 for alignment (Katoh and Standley, 2013), Gblocks v0.91b for alignment curation (Talavera and Castresana, 2007) and RAxML v7.7.2 for phylogeny inference. Trees are inferred from 1000 bootstraps (Stamatakis, 2006), all with default parameters.

Pairwise *t*-test and pairwise wilcoxon test, both with holm correction, have been performed using the R Software (R Core Team, 2014, http://www.R-project.org/) with *p*-values cut-off fixed at 0.01.

## Author contributions

FM and CV designed the study. CP performed the clustering and secretome analyses. EM designed the bioinformatics pipeline to identify the secretomes and performed the PFAM enrichment analysis. CP and CV wrote the manuscript. FM and CV edited the manuscript. All authors commented on the manuscript before submission. All authors read and approved the final manuscript.

### Conflict of interest statement

The authors declare that the research was conducted in the absence of any commercial or financial relationships that could be construed as a potential conflict of interest.

## Supplementary material

Supplementary Data sheet 1 **Prevalence of secreted Plant Cell Wall Degradative CAZymes**.

Supplementary Data sheet 2 **Raw output from CD-HIT clustering software**.

Supplementary Data sheet 3 **Assessment of genome completeness using CEGMA score**.

Supplementary Image 1 **Correlation between Proteome size, Secretome size, and number of Small-secreted proteins within different lifestyles**. Proteome size and Secretome size are plotted with linear regression and adjusted R-squared **(A)** as well as Secretome size and number of SSPs **(B)**. Confidence interval (95%) is shown in gray.

Supplementary Image 2 **Composition in secreted PCW-degrading CAZymes**. The prevalence of 21 families of CAZymes active on plant material has been compared between 6 different fungal lifestyles including saprotrophic (white rot, brown rot, and other saprotrophs) and biotrophic (ectomycorrhizal fungi, orchid symbiont, and pathogen) fungi. Boxplots for each category and every lifestyle show median (bold horizontal line), first and third quartiles (upper and lower limit of the box) and maximum and minimum values (straight vertical lines). Different letters indicates significant differences (*p* < 0.01) using pairwise comparisons with Wilcoxon rank sum test and holm correction of *p*-values.

Supplementary Image 3 **Prevalence of secreted exoproteases and endoproteases among lifestyles**. Proteasic ability is represented as a boxplot for each lifestyles, showing median (bold horizontal line), first and third quartiles (upper and lower limit of the box) and maximum and minimum values (straight vertical lines).

Supplementary Image 4 **Prevalence of secreted GX and GGGW class liapses among lifestyles**. Lipasic ability is represented as a boxplot for each lifestyles, showing median (bold horizontal line), first and third quartiles (upper and lower limit of the box) and maximum and minimum values (straight vertical lines).

## References

[B1] Alfaro M. Oguiza J. A. Ramírez L. Pisabarro A. G. (2014). Comparative analysis of secretomes in basidiomycete fungi. J. Proteomics 102, 28–43. 10.1016/j.jprot.2014.03.001 24631824

[B2] Altschul S. F. Gish W. Miller W. Myers, E. W. Lipman D. J. (1990). Basic local alignment search tool. J. Mol. Biol. 215, 403–410. 2231712 10.1016/S0022-2836(05)80360-2

[B3] Baccelli I. Lombardi L. Luti S. Bernardi R. Picciarelli P. Scala A.. (2014). Cerato-platanin induces resistance in Arabidopsis leaves through stomatal perception, overexpression of salicylic acid- and ethylene-signalling genes and camalexin biosynthesis. PLoS ONE 9:e100959. 10.1371/journal.pone.0100959 24968226 PMC4072723

[B4] Blümke A. Falter C. Herrfurth C. Sode B. Bode R. Schäfer W.. (2014). Secreted fungal effector lipase releases free fatty acids to inhibit innate immunity-related callose formation during wheat head infection. Plant Physiol. 165, 346–358. 10.1104/pp.114.236737 24686113 PMC4012593

[B5] Bonazza K. Gaderer R. Neudl S. Przylucka A. Allmaier G. Druzhinina I. S.. (2015). The fungal cerato-platanin protein EPL1 forms highly ordered layers at hydrophobic/hydrophilic interfaces. Soft Matter 11, 1723–1732. 10.1039/C4SM02389G 25599344

[B6] Bonfante P. Genre A. (2010). Mechanisms underlying beneficial plant-fungus interactions in mycorrhizal symbiosis. Nat. Commun. 1, 48. 10.1038/ncomms1046 20975705

[B7] Brouwer H. Coutinho P. M. Henrissat B. de Vries R. P. (2014). Carbohydrate-related enzymes of important Phytophthora plant pathogens. Fungal Genet. Biol. 72, 192–200. 10.1016/j.fgb.2014.08.011 25192612

[B8] Bryant M. K. Schardl C. L. Hesse U. Scott B. (2009). Evolution of a subtilisin-like protease gene family in the grass endophytic fungus Epichloë festucae. BMC Evol. Biol. 9:168. 10.1186/1471-2148-9-168 19615101 PMC2717940

[B9] Chaparro J. M. Sheflin A. M. Manter D. K. Vivanco J. M. (2012). Manipulating the soil microbiome to increase soil health and plant fertility. Biol. Fertil. Soils 48, 489–499. 10.1007/s00374-012-0691-4

[B10] Chisholm S. T. Coaker G. Day B. Staskawicz B. J. (2006). Host-microbe interactions: shaping the evolution of the plant immune response. Cell 124, 803–814. 10.1016/j.cell.2006.02.008 16497589

[B11] Chu F. H. Wang S. Y. Lee L. C. Shaw J. F. (2008). Identification and characterization of a lipase gene from Antrodia cinnamomea. Mycol. Res. 112, 1421–1427. 10.1016/j.mycres.2008.06.006 18652894

[B12] de Oliveira A. L. Gallo M. Pazzagli L. Benedetti C. E. Cappugi G. Scala A. (2011). The structure of the elicitor cerato-platanin (CP), the first member of the CP fungal protein family, reveals a double psi-barrel fold and carbohydrate binding. J. Biol. Chem. 286, 17560–17568. 10.1074/jbc.M111.223644 21454637 PMC3093830

[B13] Doré J. Perraud M. Dieryckx C. Kohler A. Morin E. Henrissat B.. (2015). Comparative genomics, proteomics and transcriptomics give new insight into the exoproteome of the basidiomycete *Hebeloma cylindrosporum* and its involvement in ectomycorrhizal symbiosis. New Phytol. 208, 1169–1187. 10.1111/nph.13546 26171947

[B14] Eastwood D. C. Floudas D. Binder M. Majcherczyk A. Schneider P. Aerts A.. (2011). The plant cell wall-decomposing machinery underlies the functional diversity of forest fungi. Science 333, 762–765. 10.1126/science.1205411 21764756

[B15] Emanuelsson O. Nielsen H. Brunak S. von Heijne G. (2000). Predicting subcellular localization of proteins based on their N-terminal amino acid sequence. J. Mol. Biol. 300, 1005–1016. 10.1006/jmbi.2000.3903 10891285

[B16] Essig A. Hofmann D. Münch D. Gayathri S. Künzler M. Kallio P. T.. (2014). Copsin, a novel peptide-based fungal antibiotic interfering with the peptidoglycan. J. Biol. Chem. 289, 34953–34964. 10.1074/jbc.M114.599878 25342741 PMC4263892

[B17] Figueiredo A. Monteiro F. Sebastiana M. (2014). Subtilisin-like proteases in plant-pathogen recognition and immune priming: a perspective. Front. Plant Sci. 5:739. 10.3389/fpls.2014.00739 25566306 PMC4271589

[B18] Finn R. D. Clements J. Eddy S. R. (2011). HMMER web server: interactive sequence similarity searching. Nucleic Acids Res. 39 (Web Server issue), W29–W37. 10.1093/nar/gkr367 21593126 PMC3125773

[B19] Floudas D. Binder M. Riley R. Barry K. Blanchette R. A. Henrissat B.. (2012). The paleozoic origin of enzymatic from 31 fungal genomes. Science 336, 1715–1719. 10.1126/science.1221748 22745431

[B20] Franklin O. Näsholm T. Högberg P. Högberg M. N. (2014). Forests trapped in nitrogen limitation - an ecological market perspective on ectomycorrhizal symbiosis. New Phytol. 203, 657–666. 10.1111/nph.12840 24824576 PMC4199275

[B21] Frey S. Lahmann Y. Hartmann T. Seiler S. Pöggeler S. (2015). Deletion of *S mgpi1* encoding a GPI-anchored protein suppresses sterility of the STRIPAK mutant ΔSmmob3 in the filamentous ascomycete *S ordaria macrospora*. Mol. Microbiol. 97, 676–697. 10.1111/mmi.13054 25989468

[B22] Garcia K. Delaux P. M. Cope K. R. Ané J. M. (2015). Molecular signals required for the establishment and maintenance of ectomycorrhizal symbioses. New Phytol. 208, 79–87. 10.1111/nph.13423 25982949

[B23] Geisseler D. Horwath W. R. Scow K. M. (2011). Soil moisture and plant residue addition interact in their effect on extracellular enzyme activity. Pedobiologia 54, 71–78. 10.1016/j.pedobi.2010.10.001

[B24] Giraldo M. C. Valent B. (2013). Filamentous plant pathogen effectors in action. Nat. Rev. Microbiol. 11, 800–814. 10.1038/nrmicro3119 24129511

[B25] Grigoriev I. V. Nikitin R. Haridas S. Kuo A. Ohm R. Otillar R.. (2014). MycoCosm portal: gearing up for 1000 fungal genomes. Nucleic Acids Res. 42, 1–6. 10.1093/nar/gkt1183 24297253 PMC3965089

[B26] Hibbett D. S. Matheny P. B. (2009). The relative ages of ectomycorrhizal mushrooms and their plant hosts estimated using Bayesian relaxed molecular clock analyses. BMC Biol. 7:13. 10.1186/1741-7007-7-13 19284559 PMC2660285

[B27] Horton P. Park K. J. Obayashi T. Fujita N. Harada H. Adams-Collier C. J.. (2007). WoLF PSORT: protein localization predictor. Nucleic Acids Res. 35, 585–587. 10.1093/nar/gkm259 17517783 PMC1933216

[B28] Hu G. Leger R. J. (2004). A phylogenomic approach to reconstructing the diversification of serine proteases in fungi. J. Evol. Biol. 17, 1204–1214. 10.1111/j.1420-9101.2004.00786.x 15525405

[B29] Huang Y. Niu B. Gao Y. Fu L. Li W. (2010). CD-HIT suite: a web server for clustering and comparing biological sequences. Bioinformatics 26, 680–682. 10.1093/bioinformatics/btq003 20053844 PMC2828112

[B30] Katoh K. Standley D. M. (2013). MAFFT multiple sequence alignment software version 7: improvements in performance and usability. Mol. Biol. Evol. 30, 772–780. 10.1093/molbev/mst010 23329690 PMC3603318

[B31] Kloppholz S. Kuhn H. Requena N. (2011). A secreted fungal effector of glomus intraradices promotes symbiotic biotrophy. Curr. Biol. 21, 1204–1209. 10.1016/j.cub.2011.06.044 21757354

[B32] Kohler A. Kuo A. Nagy L. G. Morin E. Barry K. W. Buscot F.. (2015). Convergent losses of decay mechanisms and rapid turnover of symbiosis genes in mycorrhizal mutualists. Nat. Genet. 47, 410–415. 10.1038/ng.3223 25706625

[B33] Krijger J.-J. Thon M. R. Deising H. B. Wirsel S. G. R. (2014). Compositions of fungal secretomes indicate a greater impact of phylogenetic history than lifestyle adaptation. BMC Genomics 15:722. 10.1186/1471-2164-15-722 25159997 PMC4161775

[B34] Lakshmanan V. Selvaraj G. Bais H. (2014). Functional soil microbiome: belowground solutions to an aboveground problem. Plant Physiol. 166, 689–700. 10.1104/pp.114.245811 25059708 PMC4213098

[B35] Levasseur A. Drula E. Lombard V. Coutinho P. M. Henrissat B. (2013). Expansion of the enzymatic repertoire of the CAZy database to integrate auxiliary redox enzymes. Biotechnol. Biofuels 6:41. 10.1186/1754-6834-6-41 23514094 PMC3620520

[B36] Lindahl B. D. Tunlid A. (2015). Ectomycorrhizal fungi-potential organic matter decomposers, yet not saprotrophs. New Phytol. 205, 1443–1447. 10.1111/nph.13201 25524234

[B37] Linder M. B. Szilvay G. R. Nakari-Setälä T. Penttilä M. E. (2005). Hydrophobins: the protein-amphiphiles of filamentous fungi. FEMS Microbiol. Rev. 29, 877–896. 10.1016/j.femsre.2005.01.004 16219510

[B38] Martin F. M. Kamoun S. (eds.). (2011). Effectors in plant–microbe interactions, in Effectors in Plant–Microbe Interactions (Chichester, UK: Wiley-Blackwell), i–xvi.

[B39] Martin F. Nehls U. (2009). Harnessing ectomycorrhizal genomics for ecological insights. Curr. Opin. Plant Biol. 12, 508–515. 10.1016/j.pbi.2009.05.007 19540154

[B40] Melén K. Krogh A. von Heijne G. (2003). Reliability measures for membrane protein topology prediction algorithms. J. Mol. Biol. 327, 735–744. 10.1016/S0022-2836(03)00182-7 12634065

[B41] Murphy E. Smith S. De Smet I. (2012). Small signaling peptides in Arabidopsis development: how cells communicate over a short distance. Plant Cell 24, 3198–3217. 10.1105/tpc.112.099010 22932676 PMC3462626

[B42] Muszewska A. Taylor J. W. Szczesny P. Grynberg M. (2011). Independent subtilases expansions in fungi associated with animals. Mol. Biol. Evol. 28, 3395–3404. 10.1093/molbev/msr176 21727238 PMC3247792

[B43] Nehls U. (2008). Mastering ectomycorrhizal symbiosis: the impact of carbohydrates. J. Exp. Bot. 59, 1097–1108. 10.1093/jxb/erm334 18272925

[B44] Nickel W. Rabouille C. (2009). Mechanisms of regulated unconventional protein secretion. Nat. Rev. Mol. Cell Biol. 10, 148–155. 10.1038/nrm2645 19122676

[B45] Petersen T. N. Brunak S. von Heijne G. Nielsen H. (2011). SignalP 4.0: discriminating signal peptides from transmembrane regions. Nat. Methods 8, 785–786. 10.1038/nmeth.1701 21959131

[B46] Plett J. M. Daguerre Y. Wittulsky S. Vayssières A. Deveau A. Melton S. J.. (2014). Effector MiSSP7 of the mutualistic fungus *Laccaria bicolor* stabilizes the Populus JAZ6 protein and represses jasmonic acid (JA) responsive genes. Proc. Natl. Acad. Sci. U.S.A. 111, 8299–8304. 10.1073/pnas.1322671111 24847068 PMC4050555

[B47] Plett J. M. Kemppainen M. Kale S. D. Kohler A. Legué V. Brun A.. (2011). A secreted effector protein of *Laccaria bicolor* is required for symbiosis development. Curr. Biol. 21, 1197–1203. 10.1016/j.cub.2011.05.033 21757352

[B48] Plett J. M. Martin F. (2015). Reconsidering mutualistic plant–fungal interactions through the lens of effector biology. Curr. Opin. Plant Biol. 26, 45–50. 10.1016/j.pbi.2015.06.001 26116975

[B49] Lo Presti L. Lanver D. Schweizer G. Tanaka S. Liang L. Tollot M.. (2015). Fungal effectors and plant susceptibility. Annu. Rev. Plant Biol. 66, 513–545. 10.1146/annurev-arplant-043014-114623 25923844

[B50] R Core Team (2014). R: A Language and Environment for Statistical Computing. Vienna: R Foundation for Statistical Computing.

[B51] Riley R. Salamov A. A. Brown D. W. Nagy L. G. Floudas D. Held B. W.. (2014). Extensive sampling of basidiomycete genomes demonstrates inadequacy of the white-rot/brown-rot paradigm for wood decay fungi. Proc. Natl. Acad. Sci. U.S.A. 111, 9923–9928. 10.1073/pnas.1400592111 24958869 PMC4103376

[B52] Rineau F. Roth D. Shah F. Smits M. Johansson T. Canbäck B.. (2012). The ectomycorrhizal fungus *Paxillus involutus* converts organic matter in plant litter using a trimmed brown-rot mechanism involving Fenton chemistry. Environ. Microbiol. 14, 1477–1487. 10.1111/j.1462-2920.2012.02736.x 22469289 PMC3440587

[B53] Rineau F. Shah F. Smits M. M. Persson P. Johansson T. Carleer R.. (2013). Carbon availability triggers the decomposition of plant litter and assimilation of nitrogen by an ectomycorrhizal fungus. ISME J. 1–13. 10.1038/ismej.2013.91 23788332 PMC3965319

[B54] Rovenich H. Boshoven J. C. Thomma B. P. (2014). Filamentous pathogen effector functions: of pathogens, hosts and microbiomes. Curr. Opin. Plant Biol. 20C, 96–103. 10.1016/j.pbi.2014.05.001 24879450

[B55] Ryberg M. Matheny P. B. (2012). Asynchronous origins of ectomycorrhizal clades of Agaricales. Proc. Biol. Sci. R. Soc. 279, 2003–2011. 10.1098/rspb.2011.2428 22171078 PMC3311903

[B56] Saunders D. G. O. Win J. Cano L. M. Szabo L. J. Kamoun S. Raffaele S. (2012). Using hierarchical clustering of secreted protein families to classify and rank candidate effectors of rust fungi. PLoS ONE 7:e29847. 10.1371/journal.pone.0029847 22238666 PMC3253089

[B57] Schimel J. P. Schaeffer S. M. (2012). Microbial control over carbon cycling in soil. Front. Microbiol. 3:348. 10.3389/fmicb.2012.00348 23055998 PMC3458434

[B58] Shah F. Rineau F. Canbäck B. Johansson T. Tunlid A. (2013). The molecular components of the extracellular protein-degradation pathways of the ectomycorrhizal fungus *Paxillus involutus*. New Phytol. 200, 875–887. 10.1111/nph.12425 23902518 PMC4282482

[B59] Shen H. Chen S. M. Liu W. Zhu F. He L. J. Zhang J. D.. (2015). Abolishing cell wall glycosylphosphatidylinositol-anchored proteins in candida albicans enhances recognition by Host Dectin-1. Infect. Immun. 83, 2694–2704. 10.1128/IAI.00097-15 25895969 PMC4468527

[B60] Sperschneider J. Gardiner D. M. Thatcher L. F. Lyons R. Singh K. B. Manners J. M.. (2015). Genome-wide analysis in three fusarium pathogens identifies rapidly evolving chromosomes and genes associated with pathogenicity. Genome Biol. Evol. 7, 1613–1627. 10.1093/gbe/evv092 25994930 PMC4494044

[B61] Stamatakis A. (2006). RAxML-VI-HPC: maximum likelihood-based phylogenetic analyses with thousands of taxa and mixed models. Bioinformatics 22, 2688–2690. 10.1093/bioinformatics/btl446 16928733

[B62] Stergiopoulos I. de Wit P. J. G. M. (2009). Fungal effector proteins. Annu. Rev. Phytopathol. 47, 233–263. 10.1146/annurev.phyto.112408.132637 19400631

[B63] Talavera G. Castresana J. (2007). Improvement of phylogenies after removing divergent and ambiguously aligned blocks from protein sequence alignments. Syst. Biol. 56, 564–577. 10.1080/10635150701472164 17654362

[B64] Talbot J. M. Bruns T. D. Smith D. P. Branco S. Glassman S. I. Erlandson S. (2013). Independent roles of ectomycorrhizal and saprotrophic communities in soil organic matter decomposition. Soil Biol. Biochem. 57, 282–291. 10.1016/j.soilbio.2012.10.004

[B65] Tian C. Beeson W. T. Iavarone A. T. Sun J. Marletta M. A. Cate J. H. D.. (2009). Systems analysis of plant cell wall degradation by the model filamentous fungus Neurospora crassa. Proc. Natl. Acad. Sci. U.S.A. 106, 22157–22162. 10.1073/pnas.0906810106 20018766 PMC2794032

[B66] Trejo-Hernández A. Andrade-Domínguez A. Hernández M. Encarnación S. (2014). Interspecies competition triggers virulence and mutability in Candida albicans-Pseudomonas aeruginosa mixed biofilms. ISME J. 8, 1974–1988. 10.1038/ismej.2014.53 24739628 PMC4184018

[B67] van den Brink J. de Vries R. P. (2011). Fungal enzyme sets for plant polysaccharide degradation. Appl. Microbiol. Biotechnol. 91, 1477–1492. 10.1007/s00253-011-3473-2 21785931 PMC3160556

[B68] van Ooij C. (2011). Symbiosis: establishing the roots of a relationship. Nat. Rev. Microbiol. 9, 629. 10.1038/nrmicro2642 21836620

[B69] Veneault-Fourrey C. Martin F. (2011). Mutualistic interactions on a knife-edge between saprotrophy and pathogenesis. Curr. Opin. Plant Biol. 14, 444–450. 10.1016/j.pbi.2011.03.022 21530366

[B70] Vincent D. Kohler A. Claverol S. Solier E. Joets J. Gibon J.. (2012). Secretome of the free-living mycelium from the ectomycorrhizal basidiomycete *Laccaria bicolor*. J. Proteome Res. 11, 157–171. 10.1021/pr200895f 22074047

[B71] Voigt C. A. Schäfer W. Salomon S. (2005). A secreted lipase of Fusarium graminearum is a virulence factor required for infection of cereals. Plant J. 42, 364–375. 10.1111/j.1365-313X.2005.02377.x 15842622

[B72] Wagg C. Bender S. F. Widmer F. van der Heijden M. G. A. (2014). Soil biodiversity and soil community composition determine ecosystem multifunctionality. Proc. Natl. Acad. Sci. U.S.A. 111, 5266–5270. 10.1073/pnas.1320054111 24639507 PMC3986181

[B73] Wallander H. Nilsson L. O. Hagersberg D. Baath E. (2001). Estimation of the biomass and seasonal growth of externalmycelium of ectomycorrhizal fungi in the field. New Phytol. 151, 753–760. 10.1046/j.0028-646x.2001.00199.x 33853251

[B74] Win J. Chaparro-Garcia A. Belhaj K. Saunders D. G. O. Yoshida K. Dong S.. (2012). Effector biology of plant-associated organisms: concepts and perspectives. Cold Spring Harb. Symp. Quant. Biol. 77, 235–247. 10.1101/sqb.2012.77.015933 23223409

[B75] Zerillo M. M. Adhikari B. N. Hamilton J. P. Buell C. R. Lévesque C. A. Tisserat N. (2013). Carbohydrate-active enzymes in pythium and their role in plant cell wall and storage polysaccharide degradation. PLoS ONE 8:e72572. 10.1371/journal.pone.0072572 24069150 PMC3772060

[B76] Zhao Z. Liu H. Wang C. Xu J. (2014). Correction: comparative analysis of fungal genomes reveals different plant cell wall degrading capacity in fungi. BMC Genomics 15:6. 10.1186/1471-2164-15-6 24422981 PMC3893384

