# Supplementary figures and images for: Comparative Analysis of Secretomes from Ectomycorrhizal Fungi with an Emphasis on Small-Secreted Proteins
Source: Front Microbiol. 2015 Nov 18;6:1278. doi: 10.3389/fmicb.2015.01278 (PMC4649063; doi:10.3389/fmicb.2015.01278)

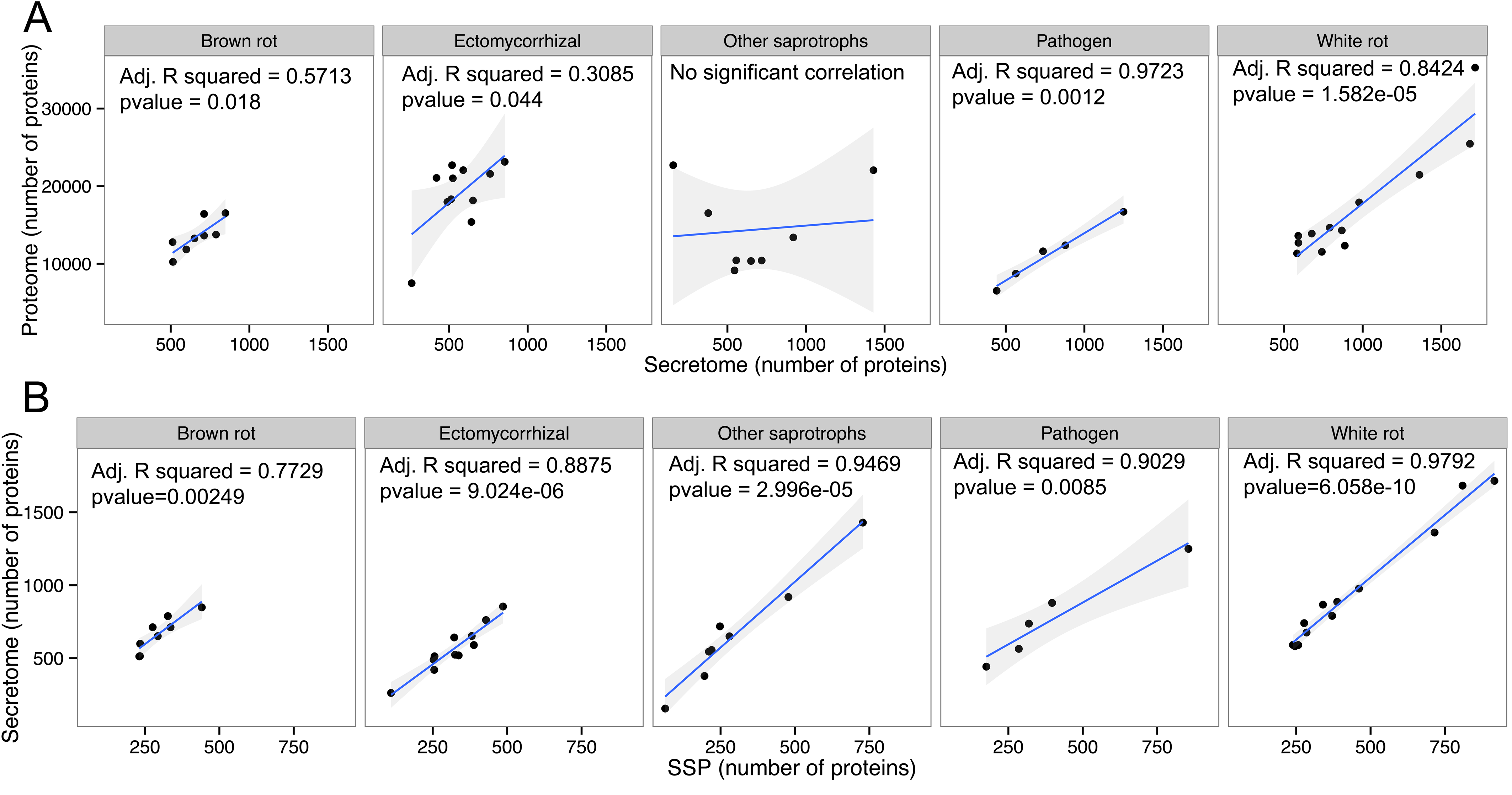

Supplement: Supplementary Image 1 — Correlation between Proteome size, Secretome size, and number of Small-secreted proteins within different lifestyles. Proteome size and Secretome size are plotted with linear regression and adjusted R-squared (A) as well as Secretome size and number of SSPs (B). Confidence interval (95%) is shown in gray. [file Image1.TIF]

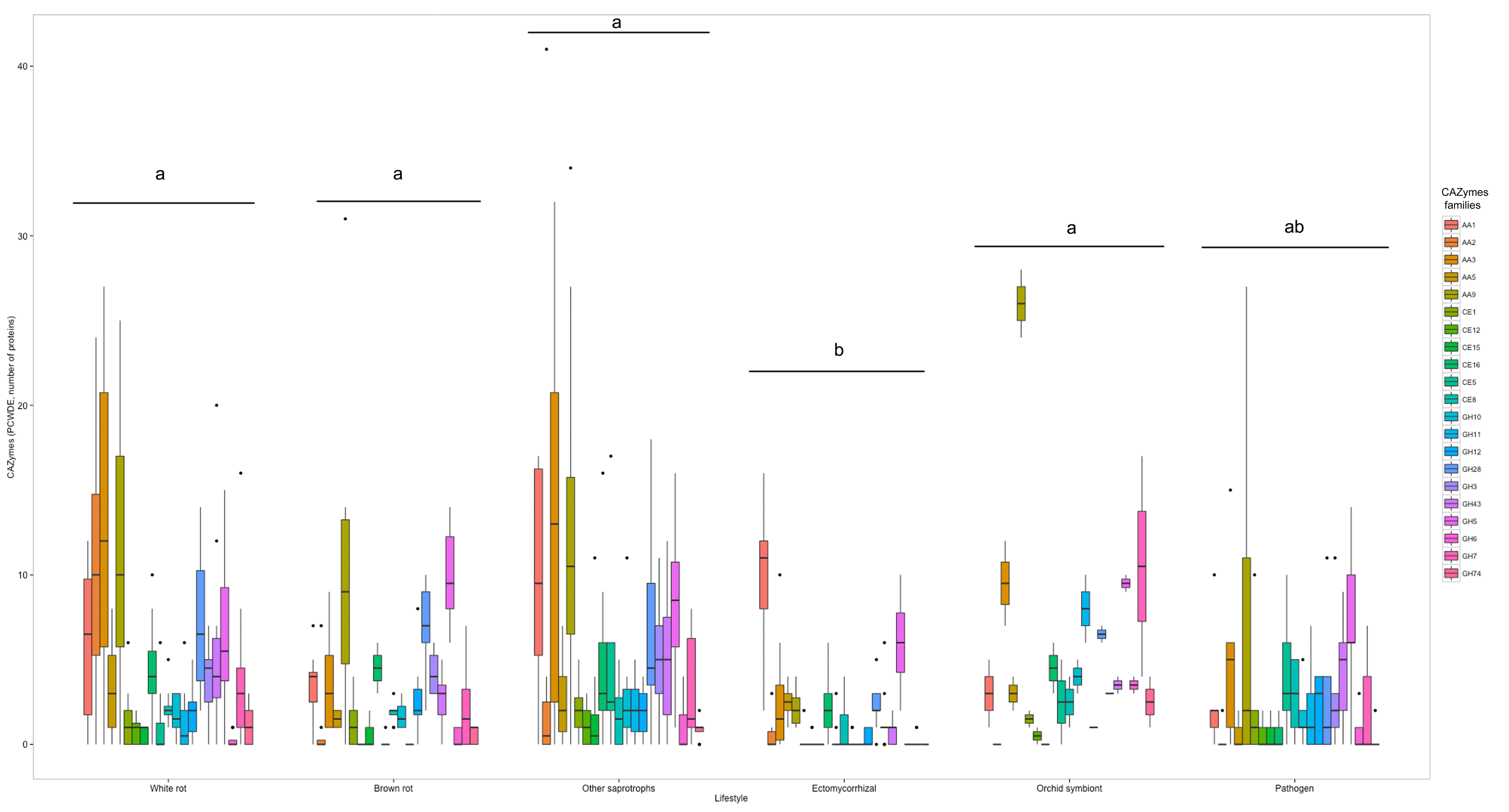

Supplement: Supplementary Image 2 — Composition in secreted PCW-degrading CAZymes. The prevalence of 21 families of CAZymes active on plant material has been compared between 6 different fungal lifestyles including saprotrophic (white rot, brown rot, and other saprotrophs) and biotrophic (ectomycorrhizal fungi, orchid symbiont, and pathogen) fungi. Boxplots for each category and every lifestyle show median (bold horizontal line), first and third quartiles (upper and lower limit of the box) and maximum and minimum values (straight vertical lines). Different letters indicates significant differences (p < 0.01) using pairwise comparisons with Wilcoxon rank sum test and holm correction of p-values. [file Image2.TIF]

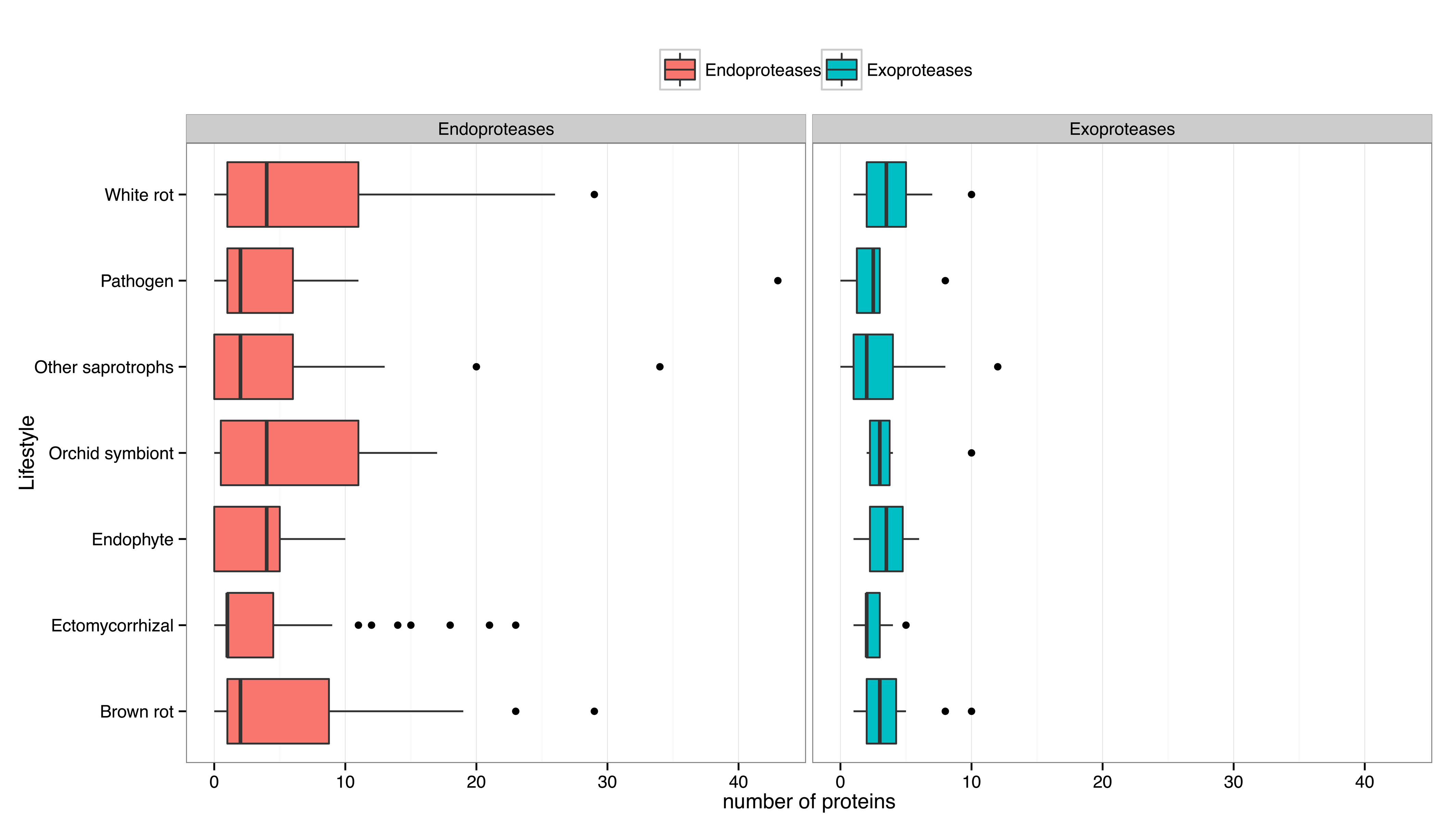

Supplement: Supplementary Image 3 — Prevalence of secreted exoproteases and endoproteases among lifestyles. Proteasic ability is represented as a boxplot for each lifestyles, showing median (bold horizontal line), first and third quartiles (upper and lower limit of the box) and maximum and minimum values (straight vertical lines). [file Image3.TIF]

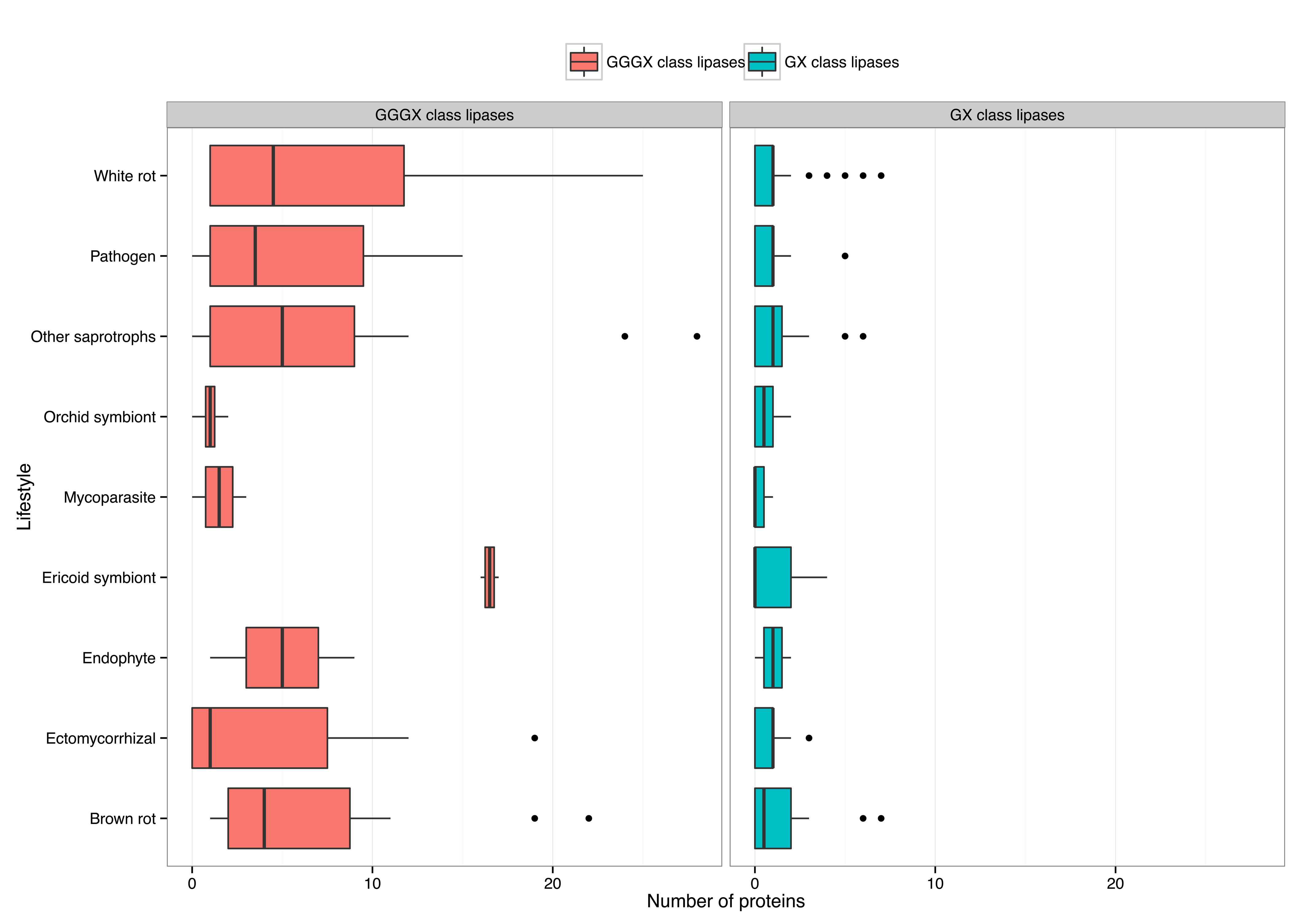

Supplement: Supplementary Image 4 — Prevalence of secreted GX and GGGW class liapses among lifestyles. Lipasic ability is represented as a boxplot for each lifestyles, showing median (bold horizontal line), first and third quartiles (upper and lower limit of the box) and maximum and minimum values (straight vertical lines). [file Image4.TIF]
